# Genome-wide prediction of CRISPR/Cas9 targets in *Kluyveromyces marxianus* and its application to obtain a stable haploid strain

**DOI:** 10.1038/s41598-018-25366-z

**Published:** 2018-05-09

**Authors:** Ming-Hsuan Lee, Jinn-Jy Lin, Yu-Ju Lin, Jui-Jen Chang, Huei-Mien Ke, Wen-Lang Fan, Tzi-Yuan Wang, Wen-Hsiung Li

**Affiliations:** 1 Doctoral Degree Program in Marine Biotechnology, National Sun Yat-sen University, 70 Lien-Hai Rd., Kaohsiung, 80424 Taiwan; 2 Doctoral Degree Program in Marine Biotechnology, Academia Sinica, 128 Academia Road, Section 2, Nankang, Taipei 11529 Taiwan; 3 Biodiversity Research Center Academia Sinica, 128 Academia Road, Sec. 2, Nankang, Taipei 115 Taiwan; 4 Biotechnology Center National Chung-Hsing University, Taichung, 40227 Taiwan; 5 Department of Medical Research China Medical University Hospital, China Medical University, No. 91 Hsueh-Shih Road, Taichung, 402 Taiwan; 6 Whole-Genome Research Core Laboratory of Human Diseases Chang Gung Medical Foundation, Maijin Road, Keelung, 222 Taiwan; 7 Department of Ecology and Evolution University of Chicago, Chicago, 60637 USA

## Abstract

*Kluyveromyces marxianus*, a probiotic yeast, is important in industrial applications because it has a broad substrate spectrum, a rapid growth rate and high thermotolerance. To date, however, there has been little effort in its genetic engineering by the CRISPR/Cas9 system. Therefore, we aimed at establishing the CRISPR/Cas9 system in *K. marxianus* and creating stable haploid strains, which will make genome engineering simpler. First, we predicted the genome-wide target sites of CRISPR/Cas9 that have been conserved among the eight sequenced genomes of *K. marxianus* strains. Second, we established the CRISPR/Cas9 system in the *K. marxianus* 4G5 strain, which was selected for its high thermotolerance, rapid growth, a pH range of pH3-9, utilization of xylose, cellobiose and glycerol, and toxin tolerance, and we knocked out its MATα3 to prevent mating-type switching. Finally, we used *K. marxianus* MATα3 knockout diploid strains to obtain stable haploid strains with a growth rate comparable to that of the diploid 4G5 strain. In summary, we present the workflow from identifying conserved CRISPR/Cas9 targets in the genome to knock out the MATα3 genes in *K. marxianus* to obtain a stable haploid strain, which can facilitate genome engineering applications.

## Introduction

*Kluyveromyces marxianus* is a yeast that can be isolated from dairy environments^1,2^. It is a probiotic yeast^3^ that is included in the list of qualified presumption of safety (QPS) biological agents by European Food Safety Authority (EFSA)^4^. It has many other advantages for a cell factory host, including a broad substrate spectrum^5^, a rapid growth rate^5,6^ and thermotolerance^6^. We have isolated a *K. marxianus* strain, called 4G5, from kifer that can grow at 48 °C, has a broad pH range (pH3-pH9), has a doubling time of 1.22 ± 0.4 hour at 30 °C in YPG (2% galactose), can utilize xylose, cellobiose, and glycerol, and can tolerate 2% isobutanol, 1.5% 1-butanol, and 4% ethanol (Supplementary Figs S1–S4 and Supplementary Table S1). These traits make it a good potential cell factory host.

The main aim of this study is to obtain stable haploid strains with a good fitness under common experimental conditions because a haploid strain can be genetically engineered more readily than a diploid strain. In response to harsh environmental conditions, *Kluyveromyces* haploids tend to become diploids and produce spores. The mating type of a cell is determined by the alleles at the MAT locus^2,7^. MATa and MATα haploids can switch their mating type^2,7^. The MATa locus includes the a1 and a2 genes, while the MATα locus includes the α1, α2 and α3 genes and α3 expresses a transposase that can fracture a MAT locus, switching MATα-type to MATa-type. The MAT locus of *K. marxianus* is homologous to that of *K. lactis*^8,9^, in which the stability of haploid cells depends on the activity of α3 transposase^10,11^. Indeed, in *K. marxianus* haploid cells, mating-type switch can happen readily and the haploid cells may become diploid cells during isolation. Thus, we aimed to knock out the MATα3 genes in the diploid background to avoid mating-type switch.

To knockout the α3 genes, we used the CRISPR/Cas9 system^12–14^. The prediction and design of guide RNAs (gRNAs) is crucial for the efficacy of CRISPR/Cas9 genome editing. There are many tools for designing gRNAs, but there are still limitations, especially for a non-model organism. First, if the genome of the strain under study is not available, as in the case of 4G5, we may need to use the available genomes of other strains to design gRNAs. However, there may be indels and nucleotide variations between the reference genome(s) and the strain under study. Second, even if one can get the same strain with the reference genome, there may be regions that are not sequenced or assembled. In either case, we propose to use the genomes of sequenced strains to identify regions conserved among the reference genomes and then to design the gRNAs in conserved regions. The designed gRNAs may also be used in future studies.

In this study, we used the above approach to obtain a stable haploid 4G5 strain, as described below. First, we assessed the evolutionary conservation of CRISPR/Cas9 targets in eight sequenced *K. marxianus* strains and optimized the gRNA targets on the Matα3 gene. Second, we established a Cas9-expressing system in *K. marxianus* 4G5 strain. Third, we integrated the gRNA cassettes of Matα3 by a modified version of PGASO (Promoter-based Gene Assembly and Simultaneous Overexpression)^15,16^ to knock out the Matα3 genes in the 4G5 genome, and obtained knockout diploid strains. Finally, we obtained stable haploid strains from the sporulation of knockout diploid strains and studied their growth rates under different temperatures and carbon sources. We selected the best strain and showed that it was stable.

## Results

### Identifying conserved regions on the *K. marxianus* genome for gRNA design

We first obtained the eight available *K. marxianus* genomes (Supplementary Table S2) and annotated their protein-coding genes (see Materials and Methods). Then we conducted the gRNA predictions and selected only gRNAs that have no computationally predicted off-targets and targeted the single-copy regions with identical sequences in these genomes. In total, we predicted 103,168 gRNAs that target the regions of 4,819 (97%) protein-coding genes that are conserved in at least two available *K. marxianus* genomes (Supplementary Table S3). In addition, we predicted 10,369 gRNAs that target the regions of 3,068 (62%) protein-coding genes that are conserved in all 8 available *K. marxianus* genomes. Based on our predicted result, we also selected gRNAs of other genes and successfully knocked out the targeted regions (data not shown) in 4G5 strain.

### Assessing the expression of Cas9 protein in transformants

**Figure 1:**
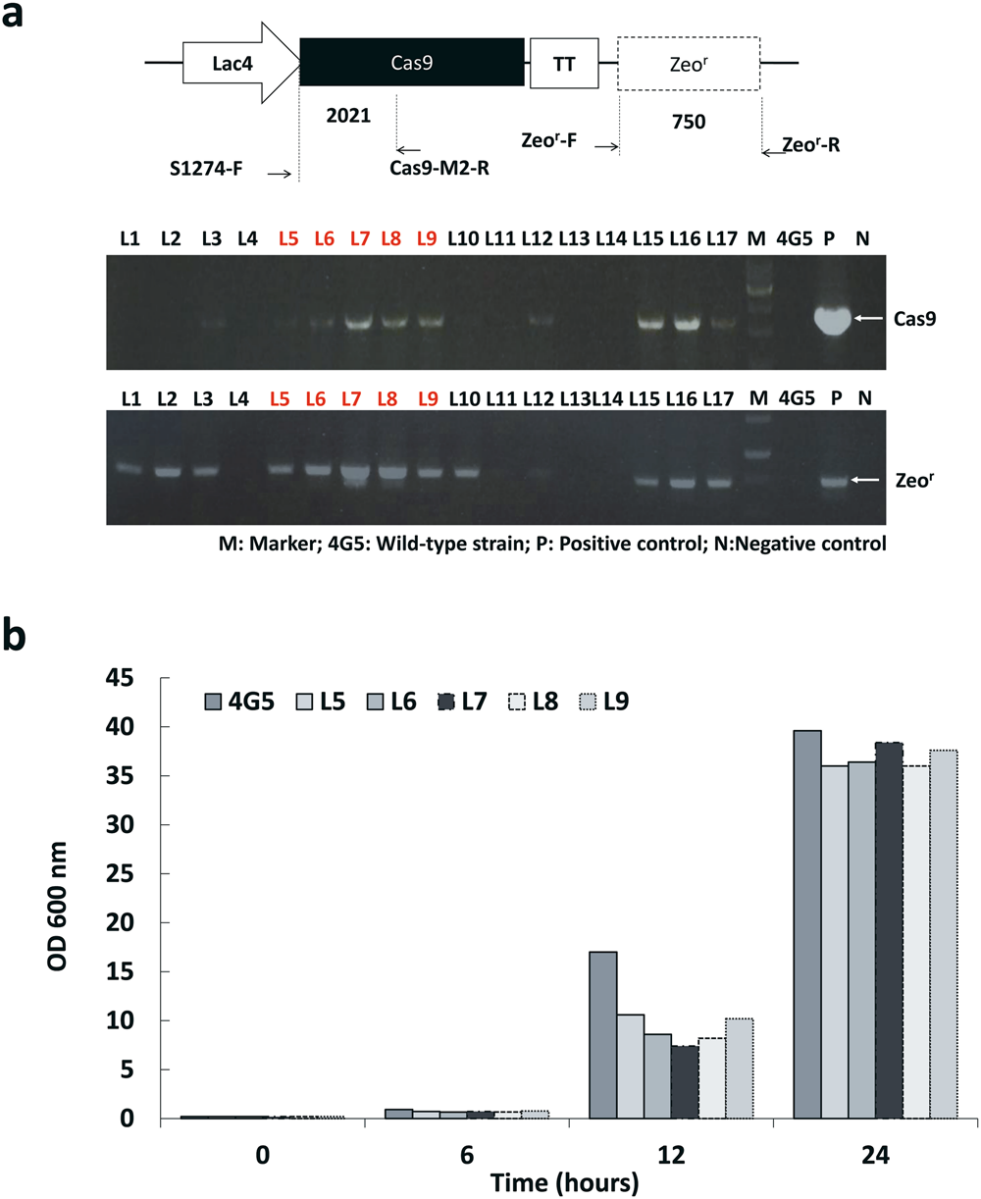
Insertion and expression of Cas9 in *Kluyveromyces marxianus* 4G5. (**a**) The transformation of the *Cas9* gene and selection marker gene (*zeocin*) was validated by PCR, confirming the two fragments of different sizes. The size of *Cas9* gene was 2021 bp, while the size of antibiotic gene (*zeocin*) was 750 bp. The five transformants L5 to L9 had the correct *Cas9* plasmid sequence (shown in the red number, Supplementary Fig. S13). (**b**) Effect of expressing Cas9 on strain growth. The growth rates of L5 to L9s were slower than the wild type 4G5 strain t 12 hours. However, the total OD_600_ of all strains were only slightly lower than 4G5 at 24 hours. The results showed that cell growth was not strongly affected by the insertion of Cas9 gene.

To do CRISPR/Cas9 genome editing, we first integrated the *Cas9* gene into the 4G5 genome via transforming the linearized *Cas9* vector (see Materials and Methods, Supplementary Fig. S5). We confirmed the success of *Cas9* gene integration in the 4G5 genome by PCR (Fig. 1a). We used two different primer pairs to check the integration of the *Cas9* gene and the z*eocin* gene (the selection marker) in the transformants, and the *Cas9* gene was found in five transformants (L5-L9) (Fig. 1a). The saturation OD_600_ of transformants could reach 30 in 24 hours, similar to the wild type, although their growth rates were slower in the first 12 hours (Fig. 1b). Therefore, the expression of Cas9 apparently did not strongly affect the growth of the transformants.

**Figure 2:**
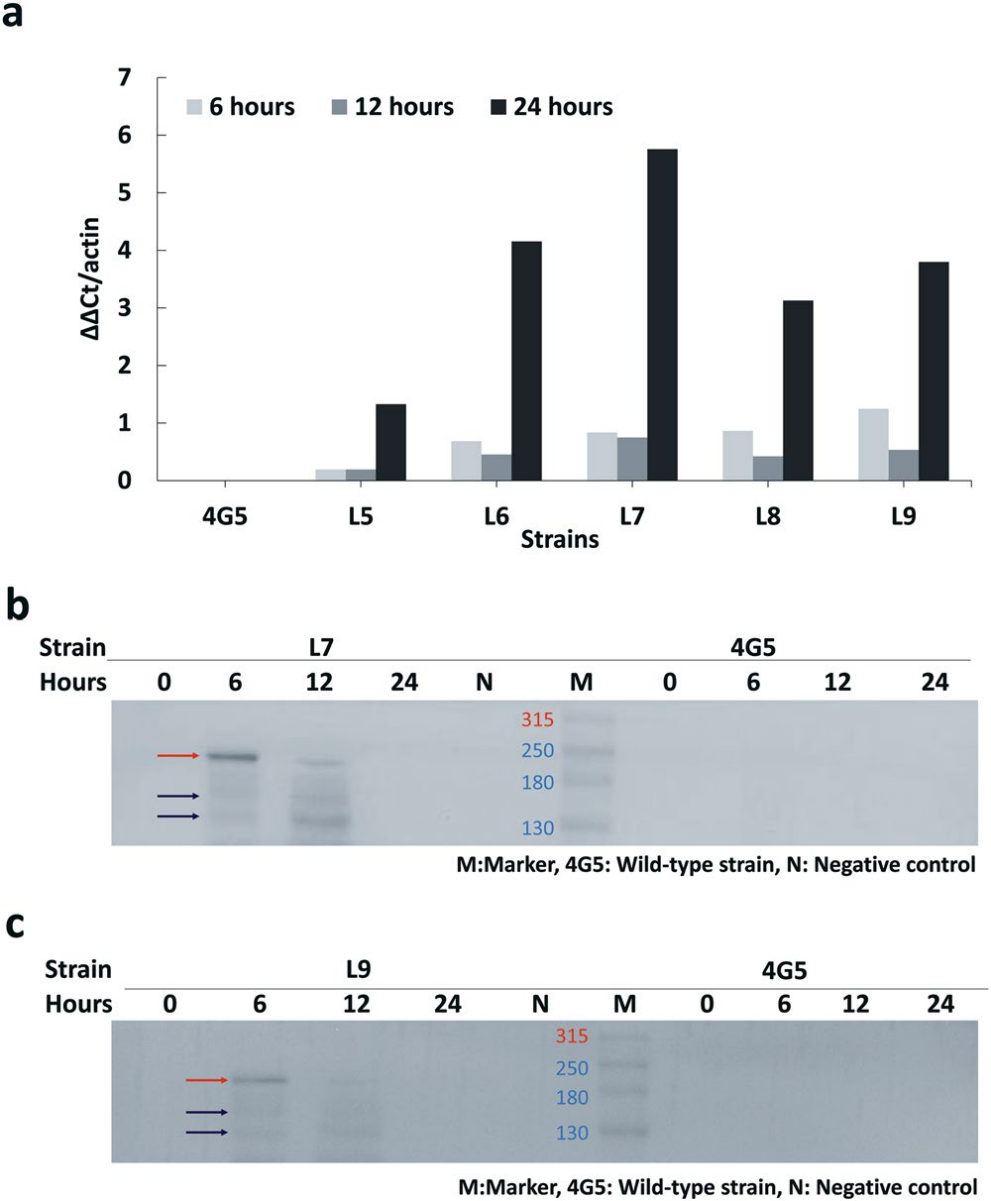
*Cas9* gene expression at different culturing times by qRT-PCR and western blot. (**a**) *Cas9* gene expression levels at different culturing times measured by qRT-PCR assays. The colors represent: gray for six hours; dark gray for 12 hours; and black for 24 hours. The strain with the highest *Cas9* gene expression at 6 hours was L9, while the strain with the highest *Cas9* gene expression at 12 hours and 24 hours was L7. (**b**) Expression profiles of Cas9 protein in L7 at different culturing times. The red and blue arrow indicates the Cas9 protein. (**c**) Expression profiles of Cas9 protein in strain L9 at different culturing times. The red (>180 kDa) and blue (<180 kDa) arrows indicate the Cas9 proteins confirmed by the LC-nESI-Q Exactive mass spectrometer (Supplementary Fig. S7).

We also investigated the *Cas9* gene copy number in each of the five transformants by quantitative PCR. The lowest copy number was found in L5, while the highest number was found in L9 (Supplementary Fig. S6). We then investigated the *Cas9* gene expression by quantitative RT-PCR at different time points (Fig. 2a) and found it expressed in all five transformants, reaching the highest expression level at 24 hours. Among the five transformants, L7 had the highest *Cas9* gene expression at 24 hours (Fig. 2a), although L9 had the highest *Cas9* gene number (Supplementary Fig. S6). We selected both L7 and L9 for *Cas9* protein qualification. The western blots of L7 and L9 strains revealed that Cas9 protein was indeed expressed and accumulated between 6 and 12 hours (Fig. 2b,c). The size of Cas9 protein was ~180 kDa, which was confirmed by the LC-nESI-Q Exactive mass spectrometer (Supplementary Fig. S7). Both *Cas9*-carrying strains were thus suitable for gRNA mutagenesis.

### CRISPR-Cas9 directed Matα3 gene knockout

To knock out the Matα3 gene, we constructed six gRNA expression cassettes (see Materials and Methods, Supplementary Table S4). We designed two gRNAs (gRNA1 and gRNA3) on the antisense strand and four gRNAs (gRNA2, gRNA4, gRNA5 and gRNA6) on the sense strand of the Matα3 gene (Supplementary Fig. S8b) by referring to the two most well-annotated genomes DMKU 3-1042 and NBRC 1777. The six gRNA target sites were conserved in most of the eight genomes we analyzed (Supplementary Fig. S9). The genomic target sites of gRNA2, gRNA4, gRNA5 and gRNA6 were at 493 bp, 157 bp, 44 bp and 22 bp downstream of the ATG start codon of the Matα3 gene on the sense strand, while those of gRNA1 and gRNA3 were located 291 bp and 382 bp downstream of the ATG start codon on the antisense strand (Table 1 and Supplementary Fig. S9).

**Table 1:** gRNAs for Matα3 gene knockout.

ID	Downstream of the ATG start codon	Guide sequences
-gRNA1	291	CAGCATTGTATTTGCTAAATGGG
gRNA2	513	ACTGGATACATCTTTGTGAGAGG
-gRNA3	328	GATATTTTTTCATTAAGAAAGGG
gRNA4	177	AAGAATTGGCTTTTGAAAAACGG
gRNA5	64	GATCCTTTGAATTTGAAGAGAGG
gRNA6	42	AAATGGATAATCCATCCAAAAGG

^*^gRNAs that facilitated the cleavage are underlined. gRNAs that target the antisense strand are indicated by “−” in their ID.

**Figure 3:**
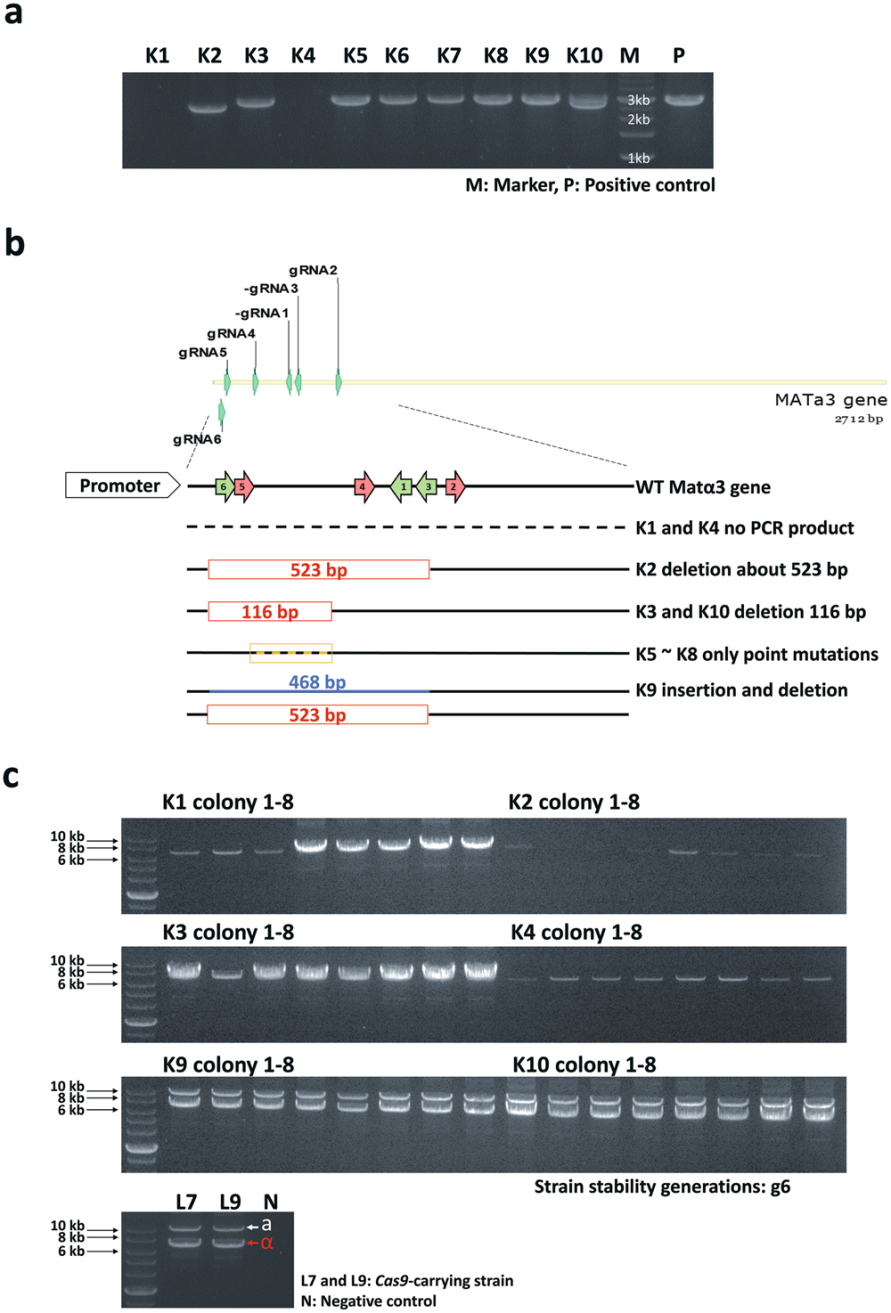
The Matα3 gene knockout transformants and validation of mating-type. (**a**) The transformants of Matα3 gene knockout was confirmed by PCR. (**b**) The success of CRISPR/Cas9 cleavage on Matα3 gene in transformants K2, K3, K9 and K10 were validated by sequencing. K1 and K4 did not produce any PCR product. K5 to K8 had only point mutations on Matα3 (Supplementary Fig. S12). Based on the location of indels, we inferred that the gRNAs facilitated the cleavage were gRNA2, gRNA4 and gRNA5. (**c**) After purification of Matα3 gene knockout for five generations, we randomly selected eight colonies from transformants K1, K2, K3, K4, K9 and K10 for confirming the type of their MAT locus. White arrow refers to MATa-type and red arrow refers to MATα-type.

The six gRNA cassettes were linearized and co-transformed into the L7 and L9 strains. After the transformants were grown on YPG-zeocin selection medium, ten colonies (K1 to K10) were picked for confirming the order of gRNA cassettes by PCR (Supplementary Table S4, S5 and Supplementary Fig. S8c). Six of the ten transformants (K1, K2, K3, K4, K5 and K9) contained all six gRNA cassettes with the correct order, while the other four transformants only contained 2 to 5 gRNAs (Supplementary Figs S10 and S11). Among the 10 transformants, K1, K2, K3, K4, K9 and K10 had large indels in their Matα3 gene, as validated by PCR and Sanger sequencing, confirming the success of CRISPR/Cas9 cleavage on the Matα3 gene (Fig. 3a,b, Supplementary Fig. S12). K2 has a deletion of 523 bp caused by gRNA2 and gRNA5 (Fig. 3b and Supplementary Fig. S12). K3 and K10 both have a deletion of 116 bp caused by gRNA4 and gRNA5. K9 has a deletion of 523 bp caused by gRNA2 and gRNA5 and an insertion of 468 bp from a non-homologous recombination with another chromosome (Fig. 3b and Supplementary Fig. S12). We failed to amplify Matα3 gene in K1 and K4 by the designed primers from the promoter sequence, and their deletions might be caused by gRNA2 and gRNA5 that deleted fragments larger than 523 bp. These results indicated that the method may be able to co-transform six different gRNAs to knock out multiple genes at once.

### Sporulation and confirmation of the mating type in haploids

**Figure 4:**
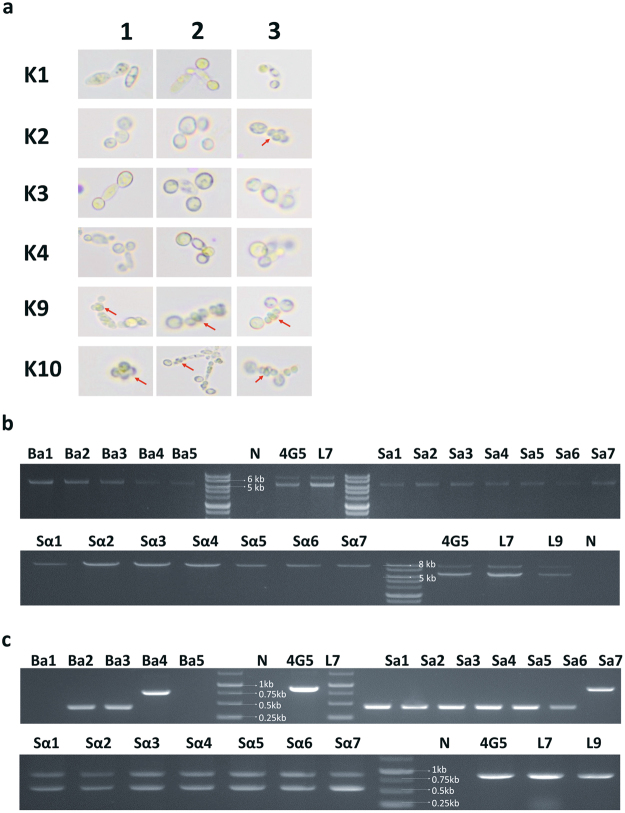
Sporulation and Matα3 gene knockout of transformants and validation of mating-type. (**a**) The haploids were obtained by sporulation experiments. The spore locations in the figure were indicated by red arrows. K1, K3 and K4 only showed budding, while K9 and K10 produced spores. One of the three K2 colonies produced spores, while the other two showed budding. (**b**) Confirmation of the MAT type of the isolated spores. In total, we isolated 12 strains of MATa-type and 7 strains of MATα-type. (**c**) Confirmation of the Matα3 gene deletion by PCR in these 12 haploid strains of MATa-type and 7 haploid strains of MATα-type.

Before obtaining haploids, we checked the MAT type of each of the six transformants of Matα3 gene-knockout mutants (K1, K2, K3, K4, K9 and K10) by PCR, using the primers designed by Lane *et al*.^2^. According to Lane *et al*.^2^, the MATα-type strain should have a 9,691 bp fragment, whereas the a-type should have a 6,652 bp fragment (Fig. 3c). The diploid should have both MAT fragments in the genome. Therefore, we confirmed the mating type of the six transformants by amplifying their MAT loci. For each transformant, we randomly selected eight colonies to check the type of the MAT loci. We found that K1, K2, K3 and K4 were all MATa-type haploids, while K9 and K10 were likely diploids (see below) (Fig. 3c). These Matα3 knockout mutants were further streaked out for 5 generations for colony purification and then cultured in nutrient deficient medium at 25 °C for 3 days for producing spores (Fig. 4a). The sporulated mutants were then selected for checking their mating type. The results showed that K1, K3 and K4 only exhibited budding and did not produce spores, whereas K9 and K10 produced spores and were classified as diploids. Thus, K1, K3 and K4 indeed were MATa-type haploids. One of the three randomly selected colonies generated from K2 had a few spores (Fig. 4a), which might be due to mating-type switch from MATa type to MATα type exerted by Kat1^17,18^. We isolated seven MATa-type (called Sa1 to Sa7) and seven MATα-type (Sα1 to Sα7) cells from sporulation of K9. Furthermore, we isolated one MATa-type haploid from only budding strain (called Ba1 strain) from K1, two MATa-type haploids (called Ba2 and Ba3 strains) from K2, one MATa-type haploid (called Ba4 strain) from K3 and one MATa-type haploid (called Ba5 strain) from K4 for comparison with the haploids isolated from sporulation. Then, we checked the MAT loci types and Matα3 gene deletion in these haploids by PCR (Fig. 4b,c). In total, 12 MATa-type haploid strains and 7 MATα-type strains were isolated and their growth rates in different carbon sources and at different temperatures were studied as described below.

### Haploid growth in different carbon sources and at different temperatures

**Figure 5:**
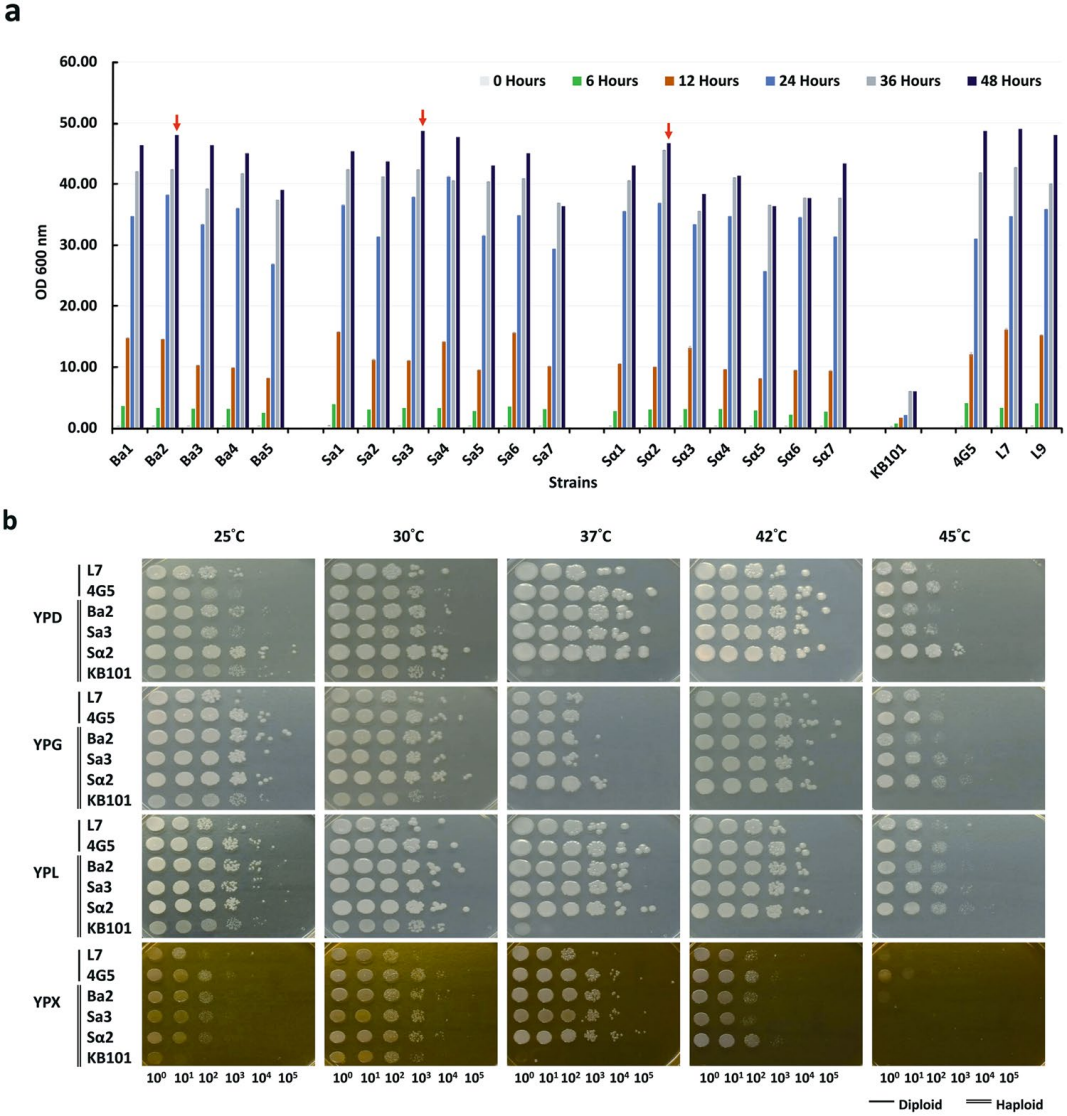
Comparison of the growth rates of haploid and diploid strains in different carbon sources and at different temperatures. (**a**) The growth rates of haploids and diploids at different culturing times. The stripe colors from light to dark represent the passage of time from 0 hour to 48 hours. The strains compared were sorted by the x-axis, which included 12 haploids of MATa-type (Ba1-Ba5 and Sa1-Sa7), 7 haploids of MATα-type (Sα1-Sα7), 3 diploids (4G5, L7 and L9) and *K. lactis* haploid KB101. Among the 21 *K. marxianus* haploids, the three strains Ka2, Sa3 and Sα2, which could achieve ~OD_600_ = 45 after 48 hours (red arrows), were selected for growth rate comparison with *K. lactis* haploid KB101 and the 4G5 and L7 diploids. (**b**) Comparison of the growth rates at different carbon sources and at different temperatures. Note that the haploids could tolerate temperature up to 45 °C.

To facilitate industrial applications, a haploid strain should retain the good characteristics of *K. marxianus* diploids, such as thermotolerance, a wide spectrum of carbon source utilization and a rapid growth rate. Therefore, we assessed the growth of the 12 MATa-type and the 7 MATα-type haploid strains under different temperatures and carbon sources. (Among these haploids, Ba2, Sa3 and Sα2 could achieve the cell density of ~OD_600_ = 45 after 48 hours culturing Fig. 5a). Their growth rates were compared with those of *K. lactis* KB101 haploid, 4G5 diploid and L7 (Fig. 5a). The comparison was conducted at different temperatures (25 °C to 45 °C) and in different carbon sources (glucose, galactose, lactose or xylose) (Fig. 5b). There was no significant difference between haploids and diploids when they were cultured at 25 °C and 30 °C. At 37 °C, all isolates grew slower in YPG medium compared to the other carbon sources. However, the haploids had higher growth rates than the diploids when galactose was the carbon source. In other carbon sources all *K. marxianus* strains showed no significant difference in growth rate. At 42 °C, no significant difference in growth rate was observed when cultured in different carbon sources, except in xylose (Fig. 5b). All strains grew slower in YPX (2% xylose) medium when the temperature was at least 42 °C and almost stopped growing at 45 °C. Note that the growth rates of the *K. marxianus* haploids and diploids were obviously better than the *K. lactis* haploid when the temperature was at least 37 °C (Fig. 5b). From these comparisons, we found that the Sα2 haploid had higher thermotolerance than the other strains in YPD and YPL (2% lactose) medium.

## Discussion

This study focused on developing a stable *K. marxianus* haploid strain for genetic engineering and industrial applications. In *Kluyveromyces*, the α3 gene plays an important role in mating-type switch from MATα-type to MATa-type, and the HMLα locus serves as DNA donor when there is the need to repair DNA damage on the MAT locus^10^. However, the α3 genes at both HMLα and MATα loci can express the α3 transposase^19^. As long as the α3 gene is retained in a diploid genome, mating type switch can occur. We showed that our method could indeed knockout the α3 genes to obtain a stable haploid.

For industrial applications, an engineered strain should retain rapid growth and utilization of multiple carbon sources. We assessed the growth rates of the 12 MATa-type and the 7 MATα-type haploid strains (Fig. 5a). From the experiments, the *Cas9*-carrying strains L7 and L9 showed no significant difference in growth than the wild type 4G5 strain. Among these haploids, Ba2, Sa3 and Sα2 could achieve the cell density of ~OD_600_ = 45 after 48 hours.

We compared the growth rates of haploids (Ba2, Sa3 and Sα2) with those of diploid (4G5 and L7) and haploid (*K. lactis* KB101) strains in different temperatures and carbon sources (Fig. 5). Our *K. marxianus* haploid strains could tolerate temperature up to 45 °C (Fig. 5b). The haploids exhibited a similar growth rate as the diploids in all carbon sources (Fig. 5b), except that the diploid strains grew much better than haploid strains at 45 °C when xylose was the carbon source (Fig. 5b). The Sα2 haploid showed better thermotolerance than the other strains in YPD and YPL media. Our experiments provided clues for how to select haploid strains under different temperatures and carbon sources. In addition, our experimental results can be combined with the strains with mating type information available to study the physiology in stress response and resource utilization of strains in different mating types and ploidies, which may facilitate obtaining haploid strains optimal for particular applications. This study demonstrates that the CRISPR/Cas9 system accelerates the screening of stable haploids from field-isolated yeasts.

## Materials and Methods

### Strains, culture media, and reagents

*K. marxianus* strain 4G5, which was isolated from kefir, was used as the host. Cells were maintained on YPG medium (1% BactoDifco-Yeast Extract, 1% BactoDifco-Peptone, and 2% Merck-galactose). For sample preparation, the cells were grown in 50 ml YPG, shaking at 250 rpm at 30 °C. *Cas9*-*zeocin* cassette transformation was conducted in YPG plate (YPG medium contains 1.5% Difco™-agar) with 200 μg/ml Zeocin^TM^ (InvivoGen, Cat. #: ant-zn-5) for selection. 200 μg/ml Hygromycin B Gold (InvivoGen, Cat. #: ant-hg-1) was used for the transformation of gRNA cassettes.

*E. coli* DH5α (RBC, Cat. #: RH619-J80) was used as the host for propagation of recombinant plasmids. It was cultivated in Luria-Bertani (LB) complete medium plate (0.5% BactoDifco-Yeast Extract, 1% BactoDifco-Tryptone, 1% NaCl, 2.4% Difco™-agar, pH 7.0) at 37 °C, with supplementation of 100 μg/ml Ampicillin (Bio Basic Inc., Ampicillin, Sodium Salt, Cat. #: 69-52-3) for selection.

### Data download

The genome sequences of eight *K. marxianus* strains (DMKU 3-1042, NBRC 1777, CBS4857, KCTC17555, B0399, DMB1, IIPE453 and UFS-Y2791) and their annotations (if available) were obtained from NCBI GenBank^20^. The details of the eight genomes are included in Supplementary Table S2.

### Designing and assessing the evolutionary conservation of CRISPR gRNA targets in *K. marxianus* genomes

Among the eight available *K. marxianus* genomes, only the genomes of NBRC1777 and DMKU 3-1042 were well annotated. Therefore, we annotated the remaining six *K. marxianus* genomes. First, the genome of DMKU3-1042^21^ was selected as the reference because it had the most complete genome assembly and the highest number of annotated genes. We then used the annotation to obtain the protein sequences of protein-coding genes in DMKU3-1042 to BLAST^22^ against the remaining seven genomes and annotated their protein-coding genes (e-value = 1e-10).

After annotating all the *K. marxianus* genomes under study, we predicted the gRNAs of all protein-coding genes in each of these genomes. Here we illustrate how we predicted the gRNAs in protein-coding genes in DMKU 3-1042. For each protein-coding gene, we used its sequence as the input of sgRNACas9^23^ (version 2.0.7) to predict the gRNAs with sequences with NGG Protospacer Adjacent Motif (PAM) and without off-target. The off-target of a gRNA is defined as a genomic site with <six mismatches to the gRNA target site. The off-target possibility was assessed using the DMKU 3-1042 genome as the background. We repeated the procedure for the other seven strains.

To assess the evolutionary conservation of CRISPR gRNA targets, we cross-compared the CRISPR gRNA targets in every *K. marxianus* gene in the eight genomes we studied. We kept only the gRNAs targets that were found in at least two genomes. In addition, we discarded the gRNAs that target regions with overlapping genes as we prefer the gRNAs that only target one gene. The complete set of predicted gRNAs is given in Supplementary Table S3.

To design the gRNAs on Matα3, we first obtained the gene sequence of Matα3 in the DMKU 3-1042^21^ and NBRC1777^24^ genomes as these two genomes were better annotated than the remaining six genomes. The genomes of DMKU 3-1042^21^ and NBRC1777^24^ were then integrated into the standalone version of CRISPOR^25^, which was then used to predict the gRNA sequences with NGG PAM by using the Matα3 gene sequence as the query. As the gene sequences of Matα3 and HMLα3 are identical, we selected the gRNAs that only target the two genes and with higher on-target efficacy. The off-target possibility was assessed using the DMKU 3-1042 and NBRC1777 genomes as the background. The on-target efficacy was assessed using CRISPOR and the scoring scheme of Doench *et al*.^26^. Finally, we selected six gRNAs for the Matα3 knockout experiments.

### *Cas9*-carrying yeast strain construction

To establish the CRISPR/Cas9 system in *K. marxianus*, we integrated the *Cas9* gene into the *K. marxianus* genome. For this purpose, we conducted the following experiments. First, the *Cas9* gene was synthesized by Zgenebio.inc (Taiwan) and constructed in RGN1-*Cas9* plasmid^27^. The commercial vector pKLAC2 (*K. lactis* Protein Expression Kit, New England Biolabs, MA) was used as the *Cas9* gene expression backbone^28^ with the *zeocin* selection marker. The *Cas9* gene was constructed using *Not*I and *Xho*I to generate the pKLAC4-*Cas9*-Zeocin plasmid (Supplementary Fig. S13), which was driven by the galactose-inducible promoter *lac4*. The correctness of plasmids was confirmed by sequencing (Supplementary Fig. S13 and Supplementary Table S6). The 5 μg DNA fragment of the pKLAC4-*Cas9*-Zeocin plasmid was linearized by *Sac*II and electroporated into *K. marxianus* 4G5 competent cells (Supplementary Fig. S5), using the protocol for *K. lactis*^29^. The transformants were selected on YPG plates containing Zeocin (200 μg/ml, InvivoGen, USA). The success of integration of *Cas9* and *zeocin* genes in the transformants was validated by primer pairs S1274-F/ Cas9-M2R, and Zeo^r^-F/Zeo^r^-R (Fig. 1a and Supplementary Table S5).

### Quantitative PCR analysis of *Cas9* gene copy number variation and RNA expression

After obtaining the *Cas9*-carrying strains, we checked the copy number variation of *Cas9* gene by quantitative PCR (qPCR), as explained below. Overnight yeast cultures were used to prepare the starting cultures with OD_600_ = 0.2 and the five *Cas9*-carrying strains were grown in YPG media at 30 °C with 300 rpm. Cells were harvested at 24 hours for *Cas9* gene copy number variation. Each genomic DNA was extracted and purified by AccuBioMed machine (AccuBioMed Co. Ltd.; iColumn 12 system; AccuPure Yeast DNA Mini Kit, Cat. #: D21096). Real-time PCR analyses were conducted with Roche LC480-II in 10 μl reaction volumes containing 2 × Roche LC480 PCR Mix, 50 ng genomic DNA (Supplementary Table S7), 1 μl each of gene-specific forward and reverse primers (5 μM) with 45 cycles of 95 °C for 15 secs and 60 °C for 1 min. The primers were designed online (https://lifescience.roche.com/en_tw/brands/universal-probe-library.html) with default setting (Supplementary Table S8). The relative copy number of *Cas9* gene was normalized to that of the *Act1* gene (ΔCt) and quantified with the ΔΔCt relative quantification method (Supplementary Fig. S6). The amplification efficiency of each primer pair was tested using 2-fold serial dilutions of the templates. The theoretical efficiency of amplification is 2.0 and the empirical amplification efficiencies of different primer pairs for *Cas9* and *Act1* were 1.96 and 2.07, respectively.

After checking the *Cas9* gene copy number, we used qPCR to check the Cas9 gene expression. This was to select better strains for applying the CRISPR/Cas9 system. The method for real-time PCR analyses was the same as we did for analyzing Cas9 gene copy number variation. Details of the gene expression for different cultured time points are described in Supplementary Text.

### Western blot analysis of expressed Cas9 protein

Among the *Cas9*-carrying strains, we picked up the strain with the highest *Cas9* gene expression and/or the strains with the highest *Cas9* gene copy number. The selected strains were grown in YPG media at 30 °C with 300 rpm. At 0 hour, 6 hours, 12 hours, and 24 hours, cells were collected for protein expression analysis (Supplementary Fig. S14 and Supplementary Table S9). For each strain, we collected 1 ml cells at each time point for Western blot analysis. The collected cells were washed by ice-water to remove the residual medium and were lysed by the lysis buffer with 20 U lyticase (SIGMA, Cat. #: L2524-10XU) for 1 hour at 37 °C. An aliquot of 50 ng cell lysate was loaded to 10% SDS-PAGE. After electrophoresis, the SDS-PAGE was transferred to PFDV membrane at 120 V and 4 °C for 105 min. The membrane was blocked in PBST (Phosphate Buffered Saline with 0.1% Tween-20) with 5% skim milk for 1 hour at room temperature and, after removing the blocking buffer, washed three times with PBST for additional 10 min. The primary antibodies of Cas9 (Cell Signaling Technology, Inc., Cas9 (7A9-3A3) mouse mAb, Cat. #: 14697) were 1000-fold diluted by fresh 0.1% PBST with 5% skim milk and added to the membrane for overnight at 4 °C. After the membrane was washed in PBST for three times, the secondary antibody (Goat Anti-Mouse IgG Secondary Antibody (HRP), Cat. #: SSA007) was 5000-fold diluted by fresh 0.1% PBST and then was added to the membrane for additional 2 hours at room temperature. Blotted membranes were imaged on a BioSpectrum 810 Imaging system.

### MS method for protein identification and analysis

The protein targets were dissected from staining protein gels with Coomassie Blue. The protocol used for trypsin digestion of proteins in gels was adapted from the method described by Wilm and Mann^30^. Details of in-gel trypsin digestion prior to LC-MS/MS analysis are described in Supplementary Text. The finally dried pellet was re-dissolved in 10–20 μl of 0.1% formic acid for LC-MS/MS analysis; For details, see Supplementary Text.

### gRNA integrated in *Cas9*-carrying yeast strain by PGASO

The six gRNA cassettes were together integrated into the selected *Cas9*-carrying strains using a modified PGASO technique^15,16^. We retained their own SNR52 promoters and SUP4 terminators in these gRNA cassettes. Used the same with PGASO cassettes promoter linker sequence (55 bp) to integrated into the *Cas9*-carrying strains genomics (Supplementary Fig. S8b). Details of guide RNA cassette construction and cassette integration into Cas9-carrying yeast strains by the modified PGASO technique are described in Supplementary Text.

### Haploid selection and growth rates in different carbon sources and temperatures

These Matα3 knockout mutants were further streaked out for 5 generations for colony purification and then cultured in nutrient deficient medium at 25 °C for 3 days for producing spores via sporulation; for details see Supplementary Text.

We compared the growth rates of candidate haploids in different carbon sources and at different temperatures, as described in Supplementary Text.

## Electronic supplementary material

Supplementary Materials

Dataset 1

Dataset 2
