# Supplementary material for: Genome-wide prediction of CRISPR/Cas9 targets in Kluyveromyces marxianus and its application to obtain a stable haploid strain
Source: Sci Rep. 2018 May 9;8:7305. doi: 10.1038/s41598-018-25366-z (PMC5943413; doi:10.1038/s41598-018-25366-z)
Supplement: Supplementary file 1 — Supplementary Materials [file 41598_2018_25366_MOESM1_ESM.docx]

**Genome-wide prediction of CRISPR/Cas9 targets in *Kluyveromyces marxianus* and its application to obtain a stable haploid strain**

**Ming-Hsuan Lee^1,2^, Jinn-Jy Lin^1^, Yu-Ju Lin^3^, Jui-Jen Chang^4^, Huei-Mien Ke^1^, Wen-Lang Fan^5^, Tzi-Yuan Wang^1^ and Wen-Hsiung Li^1,6,*^**

^1^Biodiversity Research Center, Academia Sinica, 128 Academia Road, Sec. 2, Nankang, Taipei 115, Taiwan

^2^Doctoral Degree Program in Marine Biotechnology, National Sun Yat-sen University, 70 Lienhai Rd., Kaohsiung, Taiwan

^3^Biotechnology Center, National Chung-Hsing University, Taichung, Taiwan 40227

^4^Department of Medical Research, China Medical University Hospital, China Medical University, No. 91 Hsueh-Shih Road, Taichung 402, Taiwan

^5^Whole-Genome Research Core Laboratory of Human Diseases, Chang Gung Medical Foundation, Maijin Road, Keelung222, Taiwan

^6^Department of Ecology and Evolution, University of Chicago, Chicago 60637, USA

^*^Corresponding author: whli@sinica.edu.tw

**Supplementary Text**

**Supplementary Methods**

**Quantitative RT-PCR analysis of *Cas9* expression**

After checking the *Cas9* gene copy number, we used qRT-PCR to check the Cas9 gene expression. This was to select better strains for applying the CRISPR/Cas9 system. Overnight yeast cultures were used to prepare the starting cultures with OD_600_ = 0.2 and the *Cas9*-carrying strains were grown in YPG media at 30°C with 300 rpm. At 6, 12, and 24 hours, cells were collected for gene expression analysis. Fig. 1b below shows the growth curves. Each mRNA was extracted and purified by AccBioMed machine (AccuBroMed Co. Ltd.; iColumn 12 system; AccuPure Yeast RNA Mini Kit, Cat. #: R21096) and stored at -80°C. An aliquot of 1 μg total RNA from each sample was used for cDNA synthesis (the final volume was 12 μl), and the reverse transcription was carried out with oligo-dT primers following the manufacturer’s instructions of the Super-script II kit (Invitrogen). The method for real-time PCR analyses here is the same as we did for analyzing Cas9 gene copy number variation.

**In-gel trypsin digestion**

The protein targets were dissected from staining protein gels with Coomassie Blue. The protocol used for trypsin digestion of proteins in gels was adapted from the method of Wilm and Mann [^1^](#_ENREF_1). Briefly, the protein spot from 1-D gel were manually excised and each band was cut into small pieces (~0.5 mm^3^). The gel pieces in an eppendorf tube were washed a few times with solution containing 50% methanol and 5% acetic acid for 2-3 hours, twice with solution of 25 mM NH_4_HCO_3_ in 50% acetonitril for 10 min each, and then the gel pieces were dried in a vacuum centrifuge. The reduction with DTT and alkylation with iodoacetamide of proteins in gel pieces were performed and the gel pieces were washed and dried in a vacuum centrifuge before trypsin digestion. A trypsin solution in 25 mM NH_4_HCO_3_ containing 75 to 100 ng of sequencing grade modified trypsin (Promega) in 25-40 μl was added and incubated with gel pieces for 12-16 hours at 37^o^C. To recover the tryptic peptides, a solution of 30 μl containing 5% formic acid and 50% acetonitril was added to the gel pieces, agitated in a vortex for 30-60 min and withdrawn into a new tube. The procedure was repeated with 15 μl solution. Then the two solutions were combined and dried in a vacuum centrifuge. The dried pellet was re-dissolved in 10-20 μl of 0.1% formic acid for LC-MS/MS analysis.

**MS method for protein identification and analysis**

The LC-nESI-Q Exactive mass spectrometer model from Thermo Fisher Scientific coupled with an on-line nanoUHPLC (Dionex UltiMate 3000 Binary RSLCnano) was utilized for protein identification and analysis. An Acclaim PepMap 100 C18 trap column (75 µm x 2.0 cm, 3 µm, 100 Å, Thermo Scitific) and an Acclaim PepMap RSLC C18 nano LC column (75 µm x 25 cm, 2 µm, 100 Å) were used to deliver solvent and separate tryptic peptides with a linear gradient from 5% to 35% of acetonitrile in 0.1% (v/v) formic acid for 40 min at the flow rate of 300 nl/min. The acquisition cycle of the MS data was performed on the data dependent mode with a full survey MS scan followed by 10 MS/MS scans of the top 10 precursor ions from the MS scan. The MS scan was performed with a resolving power of 70,000 over the mass-to-charge (*m/z*) ranging from 350 to 1600 and dynamic exclusion enabled. The data dependent MS/MS acquisitions were performed with 2 *m/z* isolation windows, 27% NCE, and 17,500 resolving power. Peptide and protein identification was performed using the Proteome Discoverer software (v2.1, Thermo Fisher Scientific) with SEQUEST and Mascot (v2.5, Matrix Sciences) search engines against the integrated Cas9 protein and the online *Kluyveromyces* sp. genomes with 28086 protein sequence entries. The parameters for database searches were set as follows: full trypsin digestion with 2 maximum missed cleavage sites, precursor mass tolerance = 10 ppm, fragment mass tolerance = 0.02 Da, dynamic modifications: oxidation (M), acetylation (protein N-terminal), and static modifications: carbamidomethyl (C). The identified peptides were validated using the Percolator algorithm against the decoy database search which rescored peptide spectrum matches (PSM) using q-values and posterior error probabilities. All the peptides were filtered with a q-value threshold of 0.01 (1% false discovery rate), and proteins were filter with high confidence threshold (0.01 q-value, 1% FDR).

**The guide RNA cassette construction**

We constructed the 6 guide RNA (gRNA) cassettes for the 6 selected gRNAs that targeted the Matα3 gene. The regulatory elements of the yeast gRNA expression cassette including the SNR52 promoter, multiple cloning sites, the gRNA inserting region and the SUP4 terminator were synthesized by Zgenebio.inc (Taiwan) [^2^](#_ENREF_2). The gRNAs were replaced by two-step fusion PCR with the primer pairs (Supplementary Table S4) using the gRNA of interests as the overlapping region (Supplementary Fig. S8a). All gRNAs were driven by the SNR52 promoter and terminated at the SUP4 terminator. In short, the SNR52 promoter with gRNA was amplified by PCR using the primer pair SNR52 promoter -F and M3NgX-PR (X=1-6). The SUP4 terminator with gRNA was amplified by the primer pair gRNA M3NgX-FT (X=1-6) and SUP4 terminator -R. The two fragments were then fused and amplified with the primer pair SNR52 promoter -F and SUP4 terminator -R via the overlapping gRNA region. The PCR conditions used for all gRNA fragments were as follows: 95°C for 30 secs, 30 cycles of 98°C for 10 secs, 50°C for 30 secs, and 72°C for 30 secs, with final extension at 72°C for 10 min. Each fused gRNA cassette was sub-cloned into plasmid yT&A (Yeastern Biotech, Taiwan).

**gRNA integrated in *Cas9*-carrying yeast strain by PGASO**

The 6 gRNA cassettes were together integrated into the selected *Cas9*-carrying strains using a modified PGASO technique [^3^](#_ENREF_3)^,^[^4^](#_ENREF_4). We retained their own SNR52 promoters and SUP4 terminators in these gRNA cassettes, and used the same promoter linker sequence (55bp) in the PGASO cassettes (Supplementary Figure. S8b). The two strains were grown overnight and subcultured to OD_600_= 0.5 in 50 ml YPG medium (1% BactoDifco-Yeast Extract, 1% BactoDifco-Peptone, 2% Merck-D(+)-Galactose) containing Zeocin (200 μg/ml) at 30°C, 250 rpm for additional 6-12 hours incubation. The cultured cells were harvested for competent cells preparation [^5^](#_ENREF_5). The solution containing 5 μg DNA and six guide RNA cassettes in equal molar amounts was added to 40 μl of the competent cells. The electroporation was performed as previously described [^3^](#_ENREF_3). Then, the transformed cells were recovered in 1 ml of YPD at 30°C for 16-24 hours, followed by spreading 50 μl cells on YPG with 200 μg/ml Hygromycin B. Transformants with correct assembled order of the six integrated gRNA cassettes were confirmed by PCR (Supplementary Table S5 and Supplementary Fig. S11). The thermal cycles of PCR used for the assembly of all cassettes were performed as follows: 95°C for 5 min, 30 cycles of 95°C for 30 secs, 50°C for 30 secs, and 72°C for 1 min, with final extension at 72°C for 10 min. After the transformants were grown on YPG-Zeocin selection medium, ten colonies were picked for confirming the Matα3 knockout by a pair of primers Matα3P(266bp)-F, Matα3(2.7kb)-F, Matα3(2.7kb)-R and Matα3(636bp)-R (Supplementary Table S5 and Supplementary Fig. S8c). These Matα3 knockout mutants were further streaked out for 5 generations for colony purification and then cultured in nutrient deficient medium at 25°C for 3 days for producing spores.

**Spore isolation for haploid selection**

The Matα3 knockout strains were selected to obtain spores. Three colonies of each knockout strain were inoculated in YPD for overnight, and transferred to 0.2 ml to 5 ml YEP (Yeast Extract Peptone, 2% bactopeptone, 1% yeast extract). After 8 to 12 hours, 2% potassium acetate was added for another 3 to 5 days. The cells were centrifuged for 10 secs, and washed by 1 ml H_2_O. The cells were suspended in 0.1 ml H_2_O with 10 ul Zymolyase (40 mg/ml, GRiSP, Cat. #: GK17.0100), and incubated at 30°C for 30 min. After the ascus walls were digested, the cells were washed by H_2_O, and were re-suspended in 0.5 ml H_2_O. The tube was agitated by vortex at high speed. After discarding the aqueous cell suspension, the spores adhered on the walls were then re-suspended by adding 0.01% Nonidet P-40, and sonicated on ice for 3 min. The spore suspensions were then spread on the YPD plates for haploid selection. The MAT types of all obtained spores and unsporulated candidates were validated by the primer pair Haploid-FP1 and Haploid-RP1 (Supplementary Table S5 and Supplementary Fig. S8c) designed by Lane *et al*. [^6^](#_ENREF_6)

**Haploid growth rates in different carbon sources and at different temperatures**

We compared the growth rates of candidate haploids with other *K. marxianus* diploids (4G5 isolate and *Cas9*-carrying L7 and L9 strains) and *K. lactis* haploid KB101 by culturing for 72 hours in YPG medium at 30°C. KB101 (*MATa ade trpl ura3 gal80-1*) was kindly provided by Dr. Zhenglong Gu. Candidate haploids reached similar OD_600_ of *K. marxianus* diploids at 72 hours were selected. The growth rates of selected haploids, *K. lactis* KB101 and the two diploids with higher saturated OD_600_ (L7 and 4G5) were measured for identifying carbon utility under different carbon sources (2% of glucose, galactose, lactose or xylose) and temperature sensitivity under five different temperatures (25°C, 30°C, 37°C, 42°C, or 45°C). For each strain, cells cultured for one day in 5 ml YPG medium were centrifuged and the cell pellets were washed by ice-water twice. The washed cells were suspended by water at OD_600_= 1. An aliquot of 5 μl of each 10-fold serial-diluted cells (10^0^, 10^1^, 10^2^, 10^3^, 10^4^ and 10^5^) of five candidates (Ka2, Sa3, Sα2, L7 and 4G5) was spotted on YP medium (1% BactoDifco-Yeast Extract and 1% BactoDifco-Peptone) with four different carbon sources (2%) and incubated at five temperatures for 36 hours.

**Supplementary References**

1 Shevchenko, A., Wilm, M., Vorm, O. & Mann, M. Mass spectrometric sequencing of proteins silver-stained polyacrylamide gels. *Anal Chem* **68**, 850-858 (1996).

2 DiCarlo, J. E. *et al.* Genome engineering in Saccharomyces cerevisiae using CRISPR-Cas systems. *Nucleic acids research*, gkt135 (2013).

3 Chang, J.-J. *et al.* PGASO: A synthetic biology tool for engineering a cellulolytic yeast. *Biotechnology for biofuels* **5**, 53 (2012).

4 Chang, J.-J. *et al.* Assembling a cellulase cocktail and a cellodextrin transporter into a yeast host for CBP ethanol production. *Biotechnology for biofuels* **6**, 19 (2013).

5 Beggs, J. D. Transformation of yeast by a replicating hybrid plasmid. *Nature* **275**, 104-109 (1978).

6 Lane, M. M. *et al.* Physiological and metabolic diversity in the yeast Kluyveromyces marxianus. *Antonie Van Leeuwenhoek* **100**, 507-519 (2011).

**Supplementary Tables**

**Supplementary Table S1. The average doubling time of 4G5 strain.**

| 3 replicates | DT (hour) |  | Vol. |  | mean | variance | SD  (Standard Deviation) | Min | Max |
| --- | --- | --- | --- | --- | --- | --- | --- | --- | --- |
| YPG galactose(30°C) | 1.14 |  | 5ml |  | 1.22 | 0.07 | 0.04 | 70.8 | 75.6 |
| YPG galactose(30°C) | 1.26 |  | 7ml |  | DT=( t – t. 0. )log 2/( log N - log N.0. ) | | | | |
| YPG galactose(30°C) | 1.25 |  | 50ml |  |  |  |  |  |  |

**Supplementary Table S1 is available as single .xlsx file.**

**Supplementary Table S2.** List of the 8 *Kluyveromyces marxianus* strains included in this study.

| Strain | Size (Mb) | GC% | Assembly | Level |
| --- | --- | --- | --- | --- |
| DMKU3-1042 | 10.9665 | 40.1173 | GCA_001417885.1 | Complete Genome |
| NBRC 1777 | 10.8956 | 40.117 | GCA_001417835.1 | Complete Genome |
| CBS4857 | 10.9145 | 40.1475 | GCA_001854445.1 | Complete Genome |
| B0399 | 11.5379 | 40.85 | GCA_001660455.1 | Chromosome |
| KCTC 17555 | 10.9206 | 40.2 | GCA_000299195.2 | Scaffold |
| DMB1 | 11.1654 | 40.1 | GCA_000747785.1 | Contig |
| UFS-Y2791 | 10.6955 | 40 | GCA_001692465.1 | Contig |
| IIPE453 | 10.712 | 40.2 | GCA_001466335.1 | Scaffold |

**Supplementary Table S3.** Conservation of gRNA targets in the 8 *Kluyveromyces marxianus* genomes. We listed the genes with at least 1 gRNA target conserved in the genomes of at least two *Kluyveromyces marxianus* strains. For each conserved gRNA, we listed the genomes in which it existed. The gene IDs were adapted from *Kluyveromyces marxianus* strain DMKU3-1042.

**Supplementary Table S3 is available as single .xlsx file**

**Supplementary Table S4.** Combination of gRNA cassette primers.

| **Primer** | **Sequences (5‘-3’)** |  |
| --- | --- | --- |
| SNR52 promoter-F | CGGCCTAGGTTATACAGAAACTTGA | |
| SUP4 terminator-R | CCCCATGGAGACATAAAAAACAAAA | |
| M3Ng1-PR | ATTTAGCAAATACAATGCTGGATCATTTATCTTTC | |
| M3Ng1-FT | CAGCATTGTATTTGCTAAATgttttagagct | |
| M3Ng2-PR | CTCACAAAGATGTATCCAGTGATCATTTATCTTTC | |
| M3Ng2-FT | ACTGGATACATCTTTGTGAGgttttagagct | |
| M3Ng3-PR | TTTCTTAATGAAAAAATATCGATCATTTATCTTTC | |
| M3Ng3-FT | GATATTTTTTCATTAAGAAAgttttagagctagaa | |
| M3Ng4-PR | CCGTTTTTCAAAAGCCAATTGATCATTTATCTTTC | |
| M3Ng4-FT | AATTGGCTTTTGAAAAACGGgttttagagctagaa | |
| M3Ng5-PR | CTCTTCAAATTCAAAGGATCGATCATTTATCTTTC | |
| M3Ng5-FT | GATCCTTTGAATTTGAAGAGgttttagagctagaa | |
| M3Ng6-PR | TTTGGATGGATTATCCATTTGATCATTTATCTTTC | |
| M3Ng6-FT | AAATGGATAATCCATCCAAAgttttagagctagaa | |

**Supplementary Table S5.** Gene check primers for fast screening.
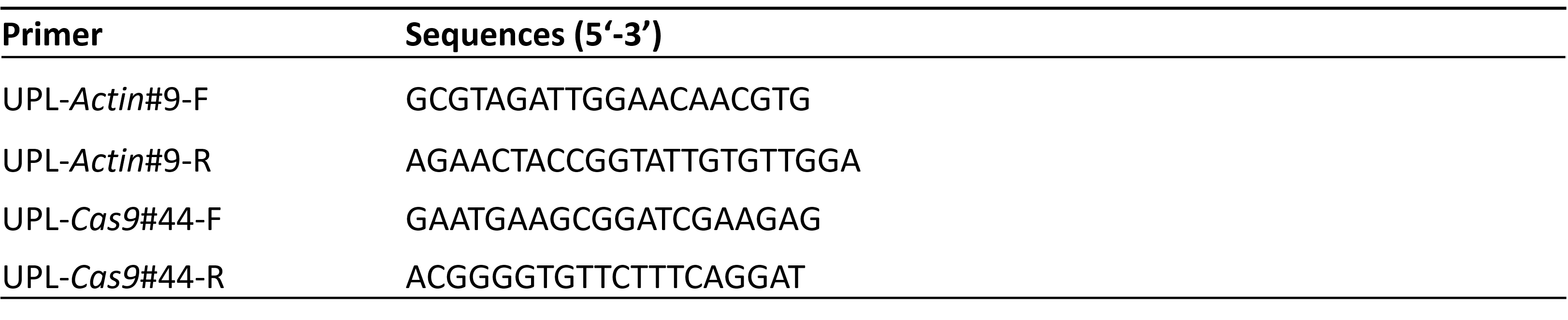

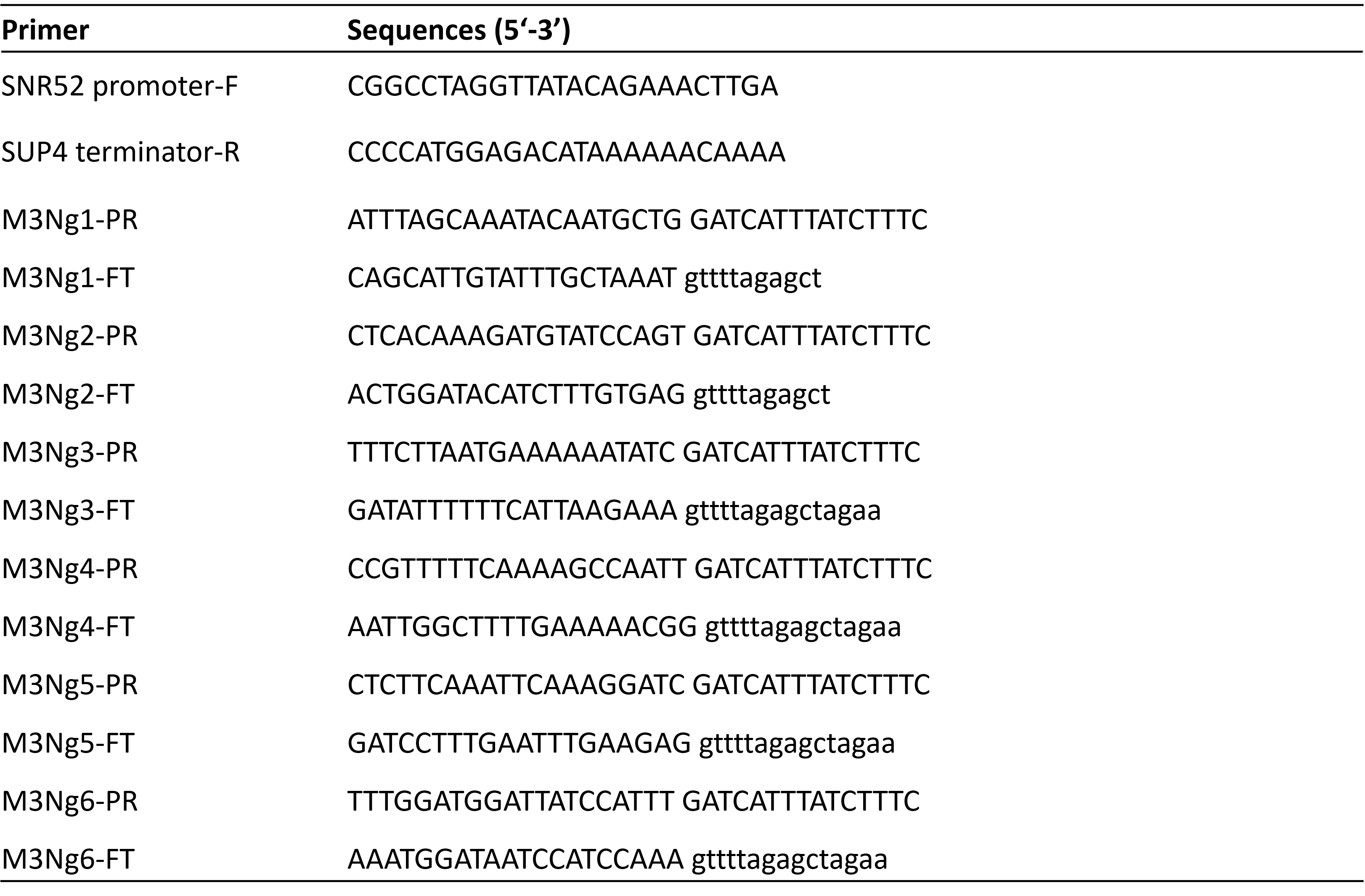


**Table 3 Combination of gRNA cassette primers.**

| **Primer** | **Sequences (5‘-3’)** |
| --- | --- |
| S1274-F | GCGGATAACAAGCTCAAC |
| Cas9-M2-R | TTCCTCGATCATCTCTCTGTCCTCA |
| Zeo^r^-F | TTCTGCACAATATTTCAAGC |
| Zeo^r^-R | GGTCGTCCAGCCTCAGGCAG |
| Matα3(2.7 kb)-F | ATGACACAAGTAGATATATCAAAAT |
| Matα3(2.7 kb)-R | TTATATAGGAAGGGGCATACTACCT |
| Matα3(636 bp)-R | AGGTTGAGGAGCAACTAAACTGT |
| Matα3P(266 bp)-F | TGGCGTCAAGCAGATTAAATGTGA |
| Haploid-FP1 | TATACATGGGATCATAAATC |
| Haploid-RP1 | CTTTGTCTTGTATGATATC |

**Supplementary Table S6.** Primers for pKLAC4-Cas9-Zeocin plasmid sequencing.

| **Primer** | **Sequences (5‘-3’)** |
| --- | --- |
| M1R | AGAACCTGTCCGACGCCATCCTGCT |
| M2R | TTCCTCGATCATCTCTCTGTCCTCA |
| M3R | AGGCGTCGTGGGCGTGGTGGTAGTT |
| M4R | TCGAAGCCGCCGTACTTCTTAGGGT |
| M5R | TCTTTCAGGATCTGGCTGCCCAGCT |
| S1276R | TCGGCACTAATAACCGTT |

**Supplementary Table S7.** Concentration of genomic DNA in Cas9-carrying strains L5-L9.

| Replicate 1 | 1 | 2 | 3 | 4 | 5 | 6 |
| --- | --- | --- | --- | --- | --- | --- |
| Strains | 4G5 | L5 | L6 | L7 | L8 | L9 |
| ΔCT | 0 | 0.32 | 0.84 | 1.09 | 1.39 | 1.33 |
| CNV |  | 1 | 2.63 | 3.42 | 4.36 | 4.17 |
|  |  |  |  |  |  |  |
| Replicate 2 | 1 | 2 | 3 | 4 | 5 | 6 |
| Strains | 4G5 | L5 | L6 | L7 | L8 | L9 |
| ΔCT | 0 | 0.49 | 1.11 | 1.87 | 1.03 | 1.96 |
| CNV |  | 1 | 2.26 | 3.8 | 2.09 | 3.99 |

**Supplementary Table S8.** qRT-PCR primers for cas9 gene expression.

| **Primer** | **Sequences (5‘-3’)** |
| --- | --- |
| UPL-*Actin*#9-F | GCGTAGATTGGAACAACGTG |
| UPL-*Actin*#9-R | AGAACTACCGGTATTGTGTTGGA |
| UPL-*Cas9*#44-F | GAATGAAGCGGATCGAAGAG |
| UPL-*Cas9*#44-R | ACGGGGTGTTCTTTCAGGAT |

**Supplementary Table S9.** Concentration of total protein in Cas9-carrying strains L7 and L9.

| Western sample name | | Hours | μg/ml |
| --- | --- | --- | --- |
| 1 | 4G5 | 0 | 702.33 |
| 2 |  | 6 | 719.00 |
| 3 |  | 12 | 849.00 |
| 4 |  | 24 | 845.67 |
| 5 | L7 | 0 | 742.33 |
| 6 |  | 6 | 684.00 |
| 7 |  | 12 | 795.67 |
| 8 |  | 24 | 812.33 |
| 9 | L9 | 0 | 684.00 |
| 10 |  | 6 | 625.67 |
| 11 |  | 12 | 779.00 |
| 12 |  | 24 | 742.33 |

**Supplementary Figures**

**
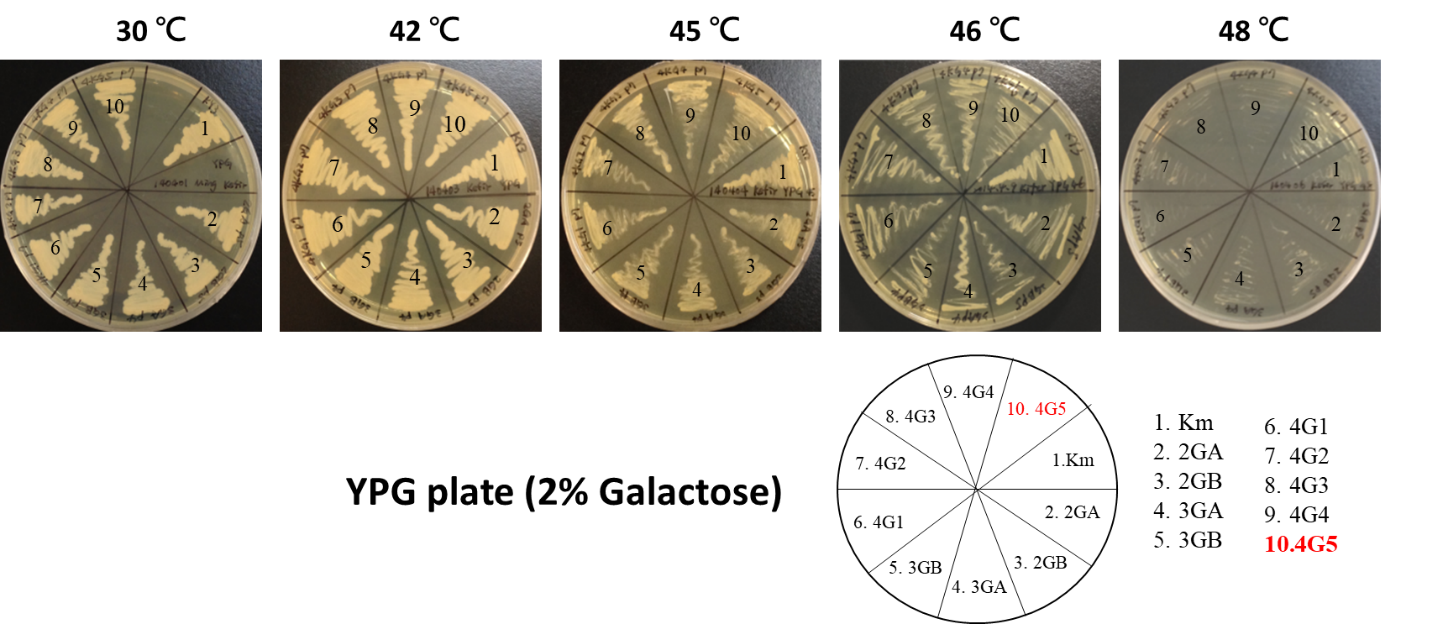
**

**Supplementary Figure S1.** Selection of the *K. marxianus* 4G5 strain. We isolated 10 *K. marxianus* strains and tested their relative growth rates on YPG plates under different temperatures. The 4G5 strain grew better than the other strains at 48 °C. Km is a *K. marxianus* strain we used previous studies, while the other strains were isolated in this study.

**
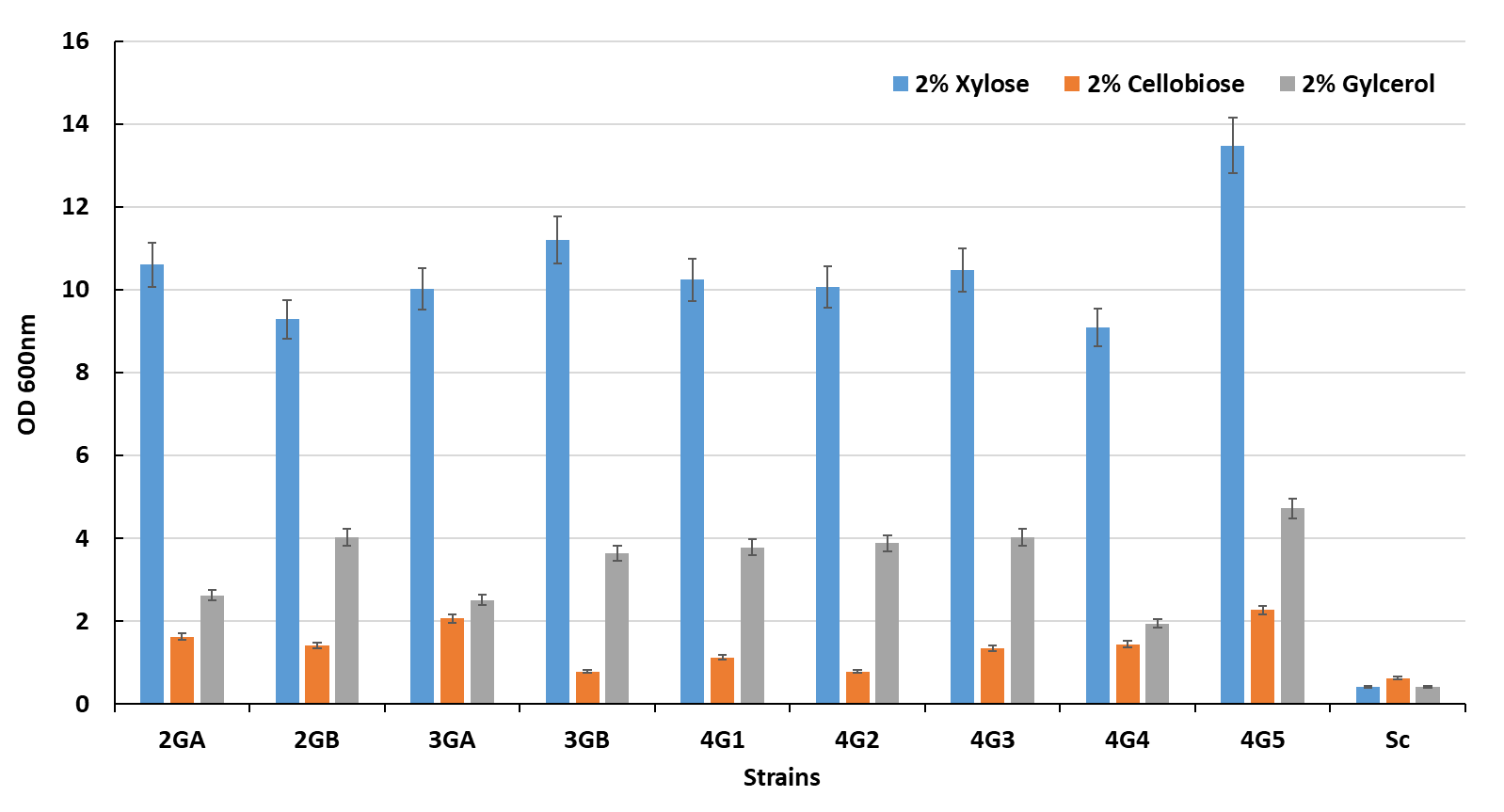
**

**Supplementary Figure S2.** Comparison of growth rates of isolated *K. marxianus* strains under three different carbon sources (2% xylose, 2% cellobiose and 2% glycerol). The data showed that the4G5 straingrew better than the other strains under different carbon sources at 65 hours of culturing. Sc is *Saccharomyces cerevisiae* and the other strains were isolated in this study.

**
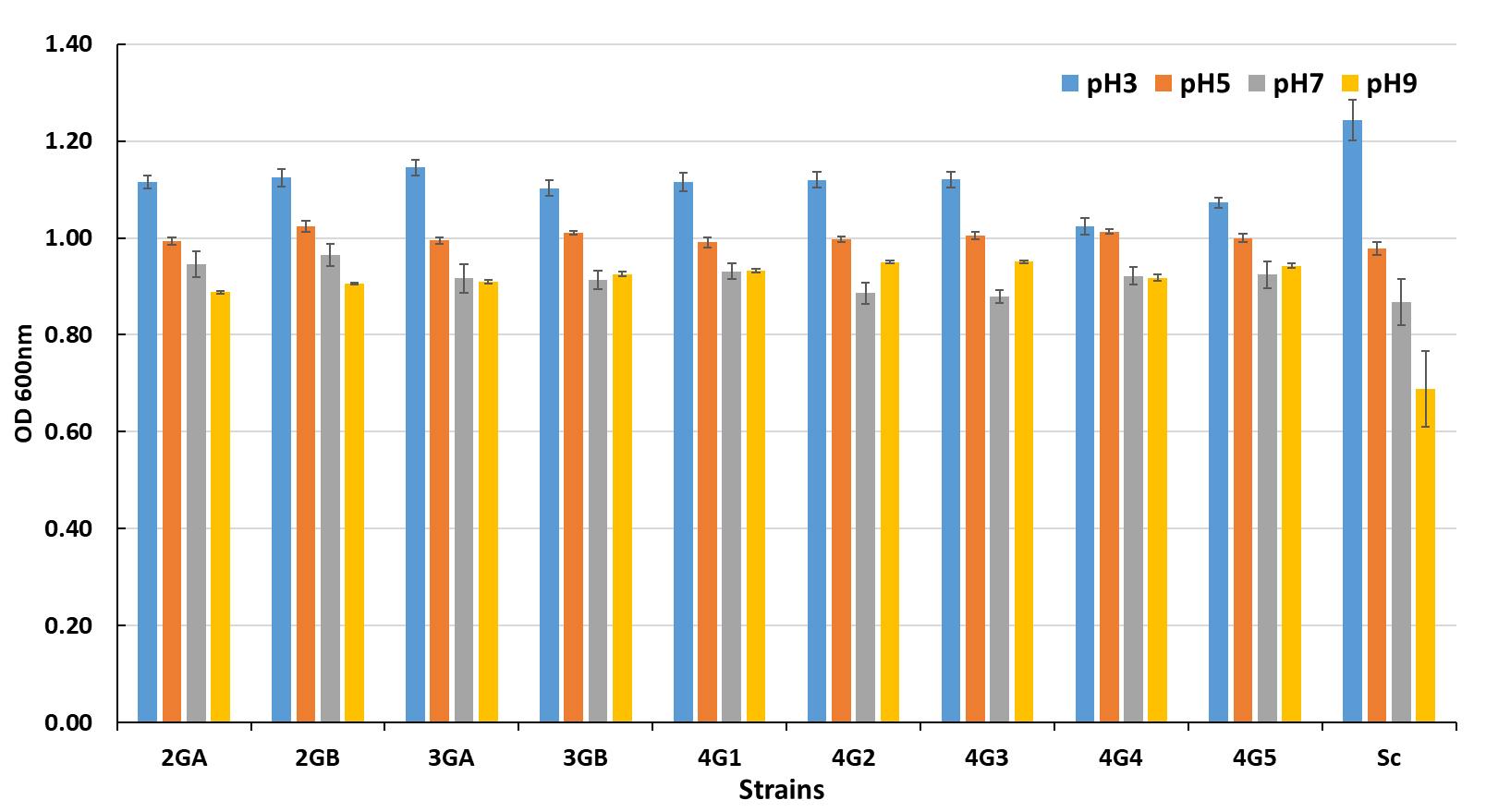
**

**Supplementary Figure S3.** Comparison of growth rates of isolated *K. marxianus* strains under different pH values at 48 hours of cultivation. The 4G5 strain as well as the other isolated K marxianus strains all have a wide pH range (pH 3-pH9). Sc is *Saccharomyces cerevisiae* and the other strains were isolated in this study.

**
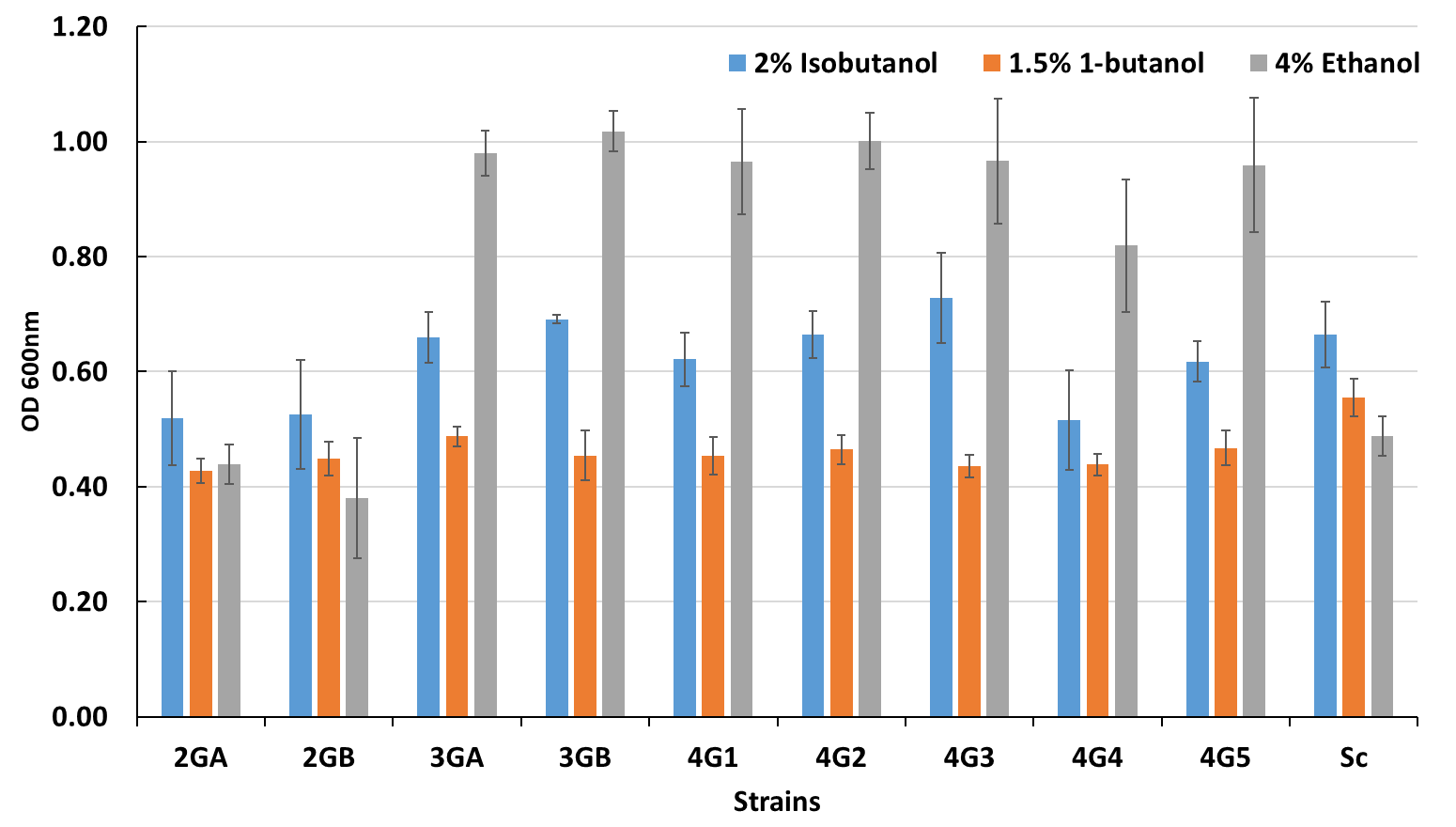
**

**Supplementary Figure S4.** Comparison of growth rates of isolated *K. marxianus* strains under different toxic substances. Our data showed that the 4G5 strain and some other strains have high toxin tolerance at 72 hours. Sc is *Saccharomyces cerevisiae* and the other strains were isolated in this study.


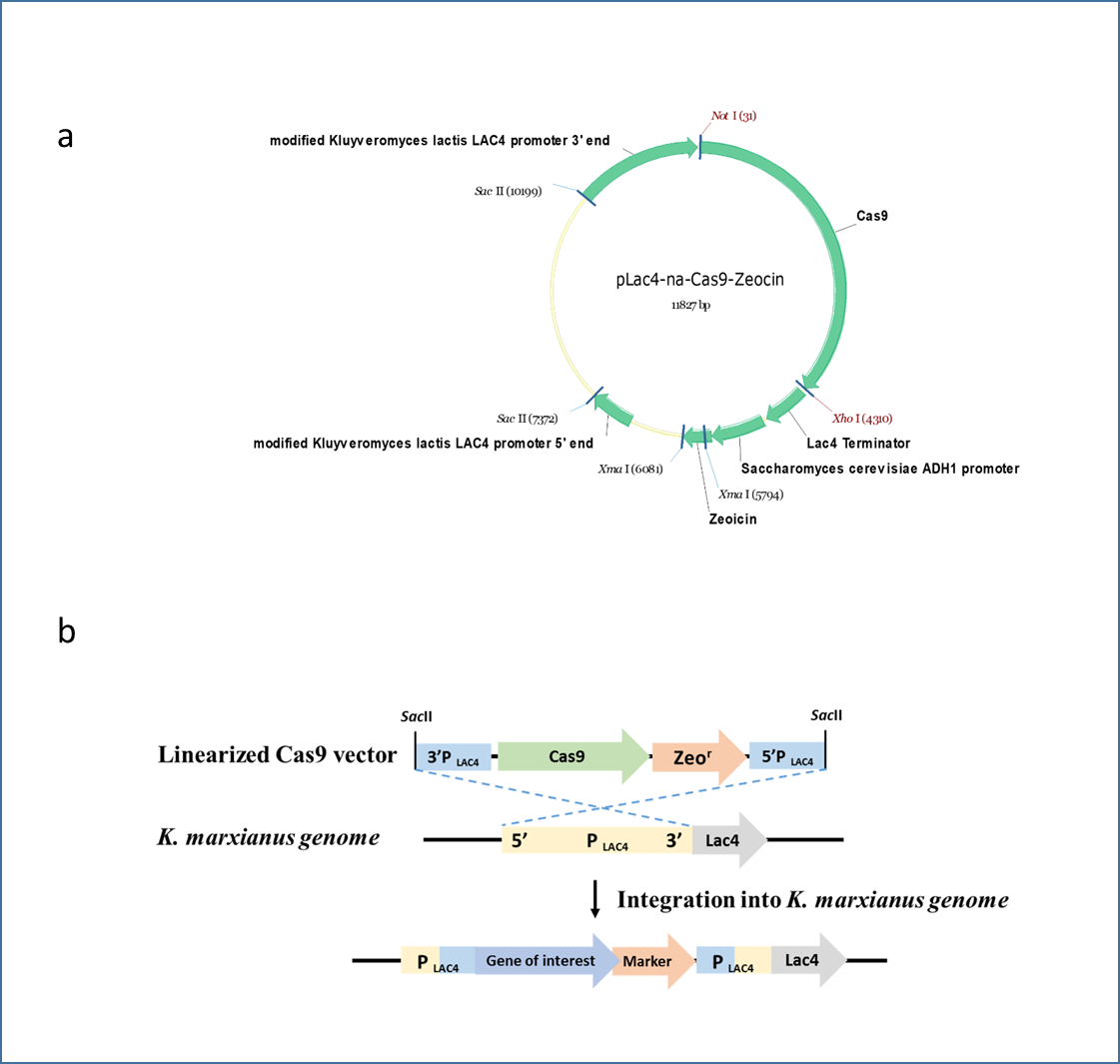


**Supplementary Figure S5.** Construction of pKLAC4-Cas9-Zeocin plasmid and PGASO gene cassettes and transformation of the linearized vector into the 4G5 genome. (a) pKLAC4-Cas9-Zeoicin map. The *Cas9* gene was digested by *Not*I and *Xho*I and ligated to the pLac4-Zeocin to generate the pKLAC4-Cas9-Zeocin plasmid, which can regulate *Cas9* gene expression by LAC4 promoter and terminator. (b) Transformation of the linearized vector into the 4G5 genome. Cas9 protein was overexpressed by the linearized vector, which was integrated into the 4G5 genome to generate a *Cas9*-carrying strain.


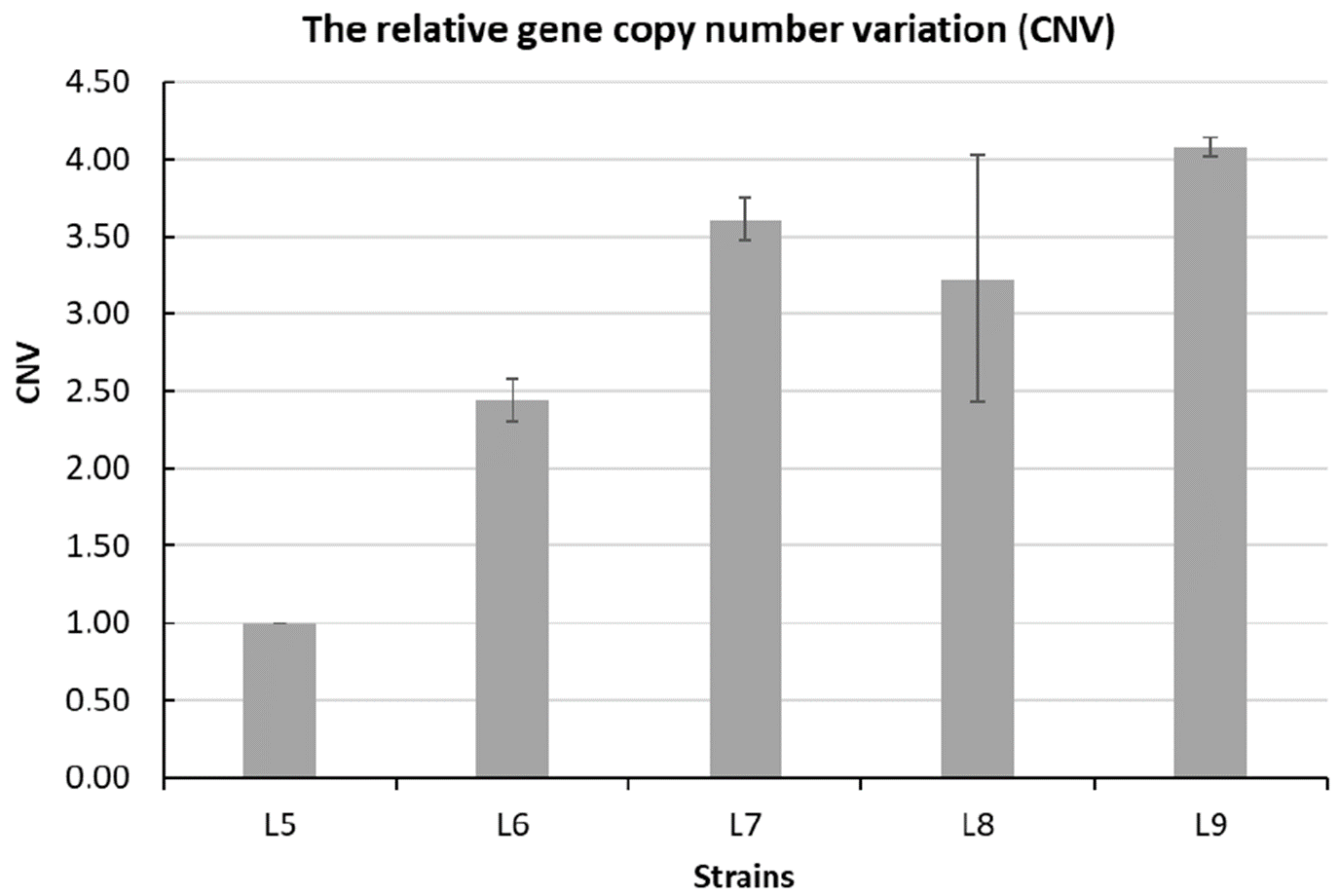


**Supplementary Figure S6.** Cas9 gene copy numbers in different strains estimated by quantitative PCR.


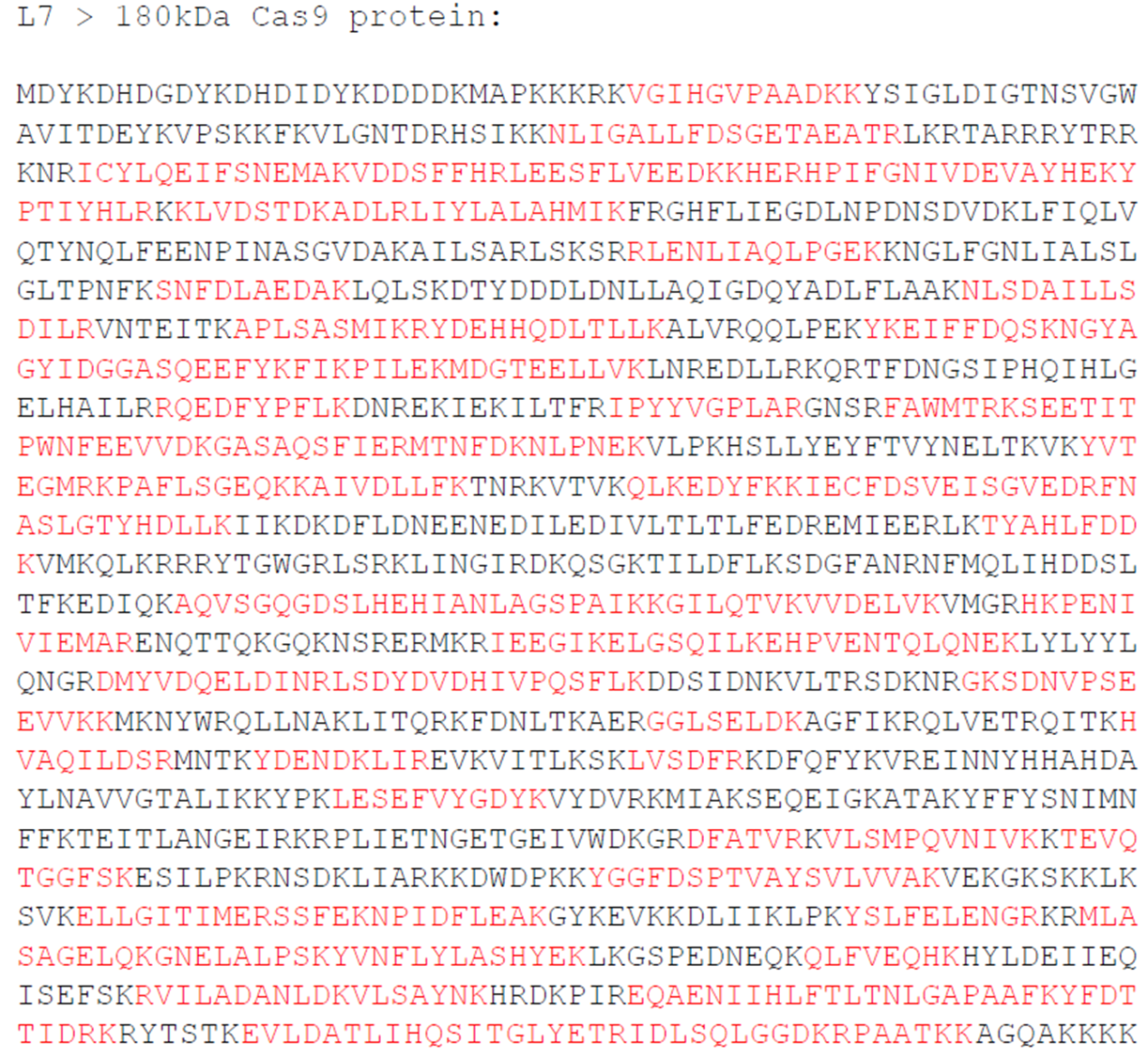


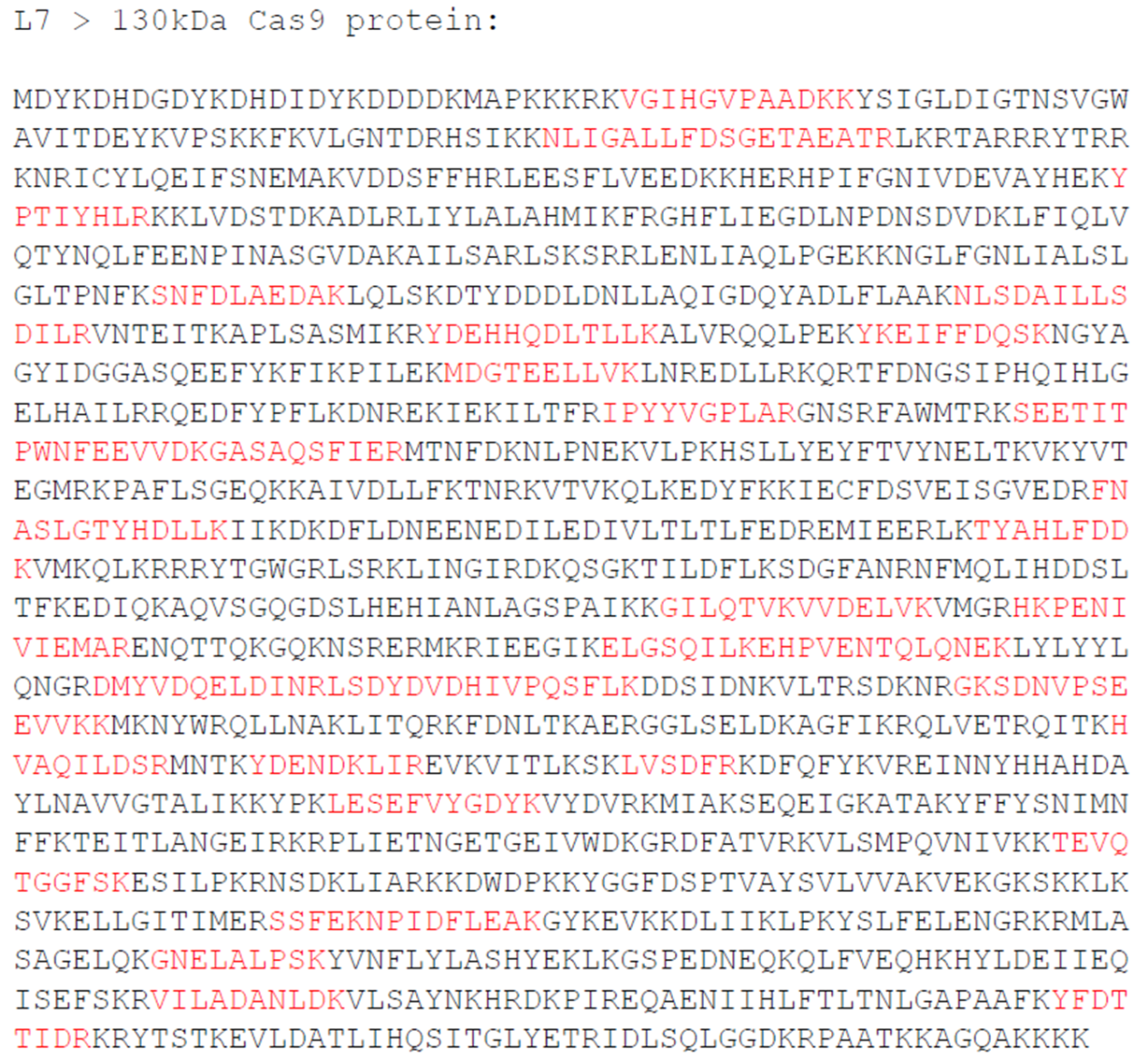


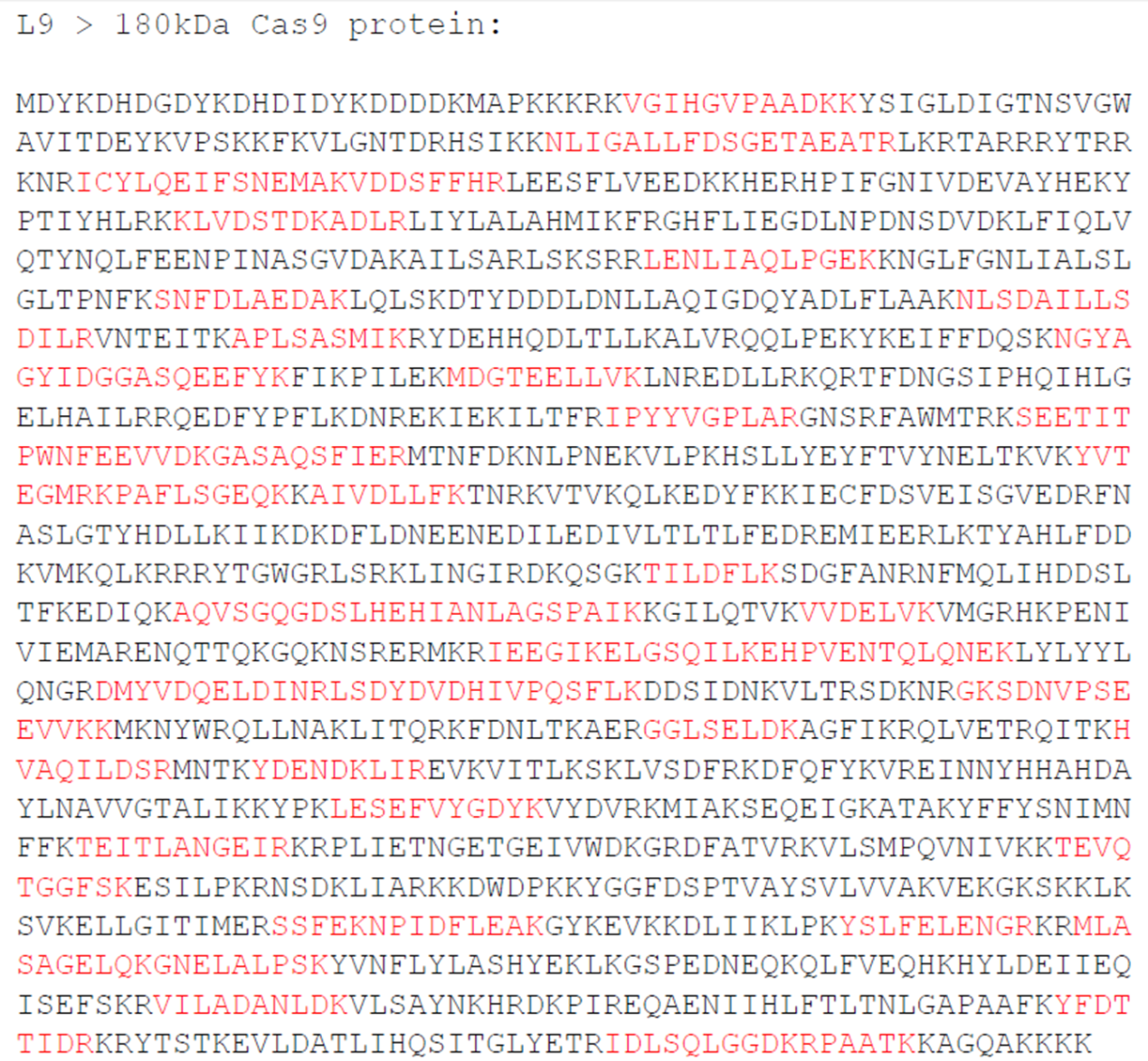


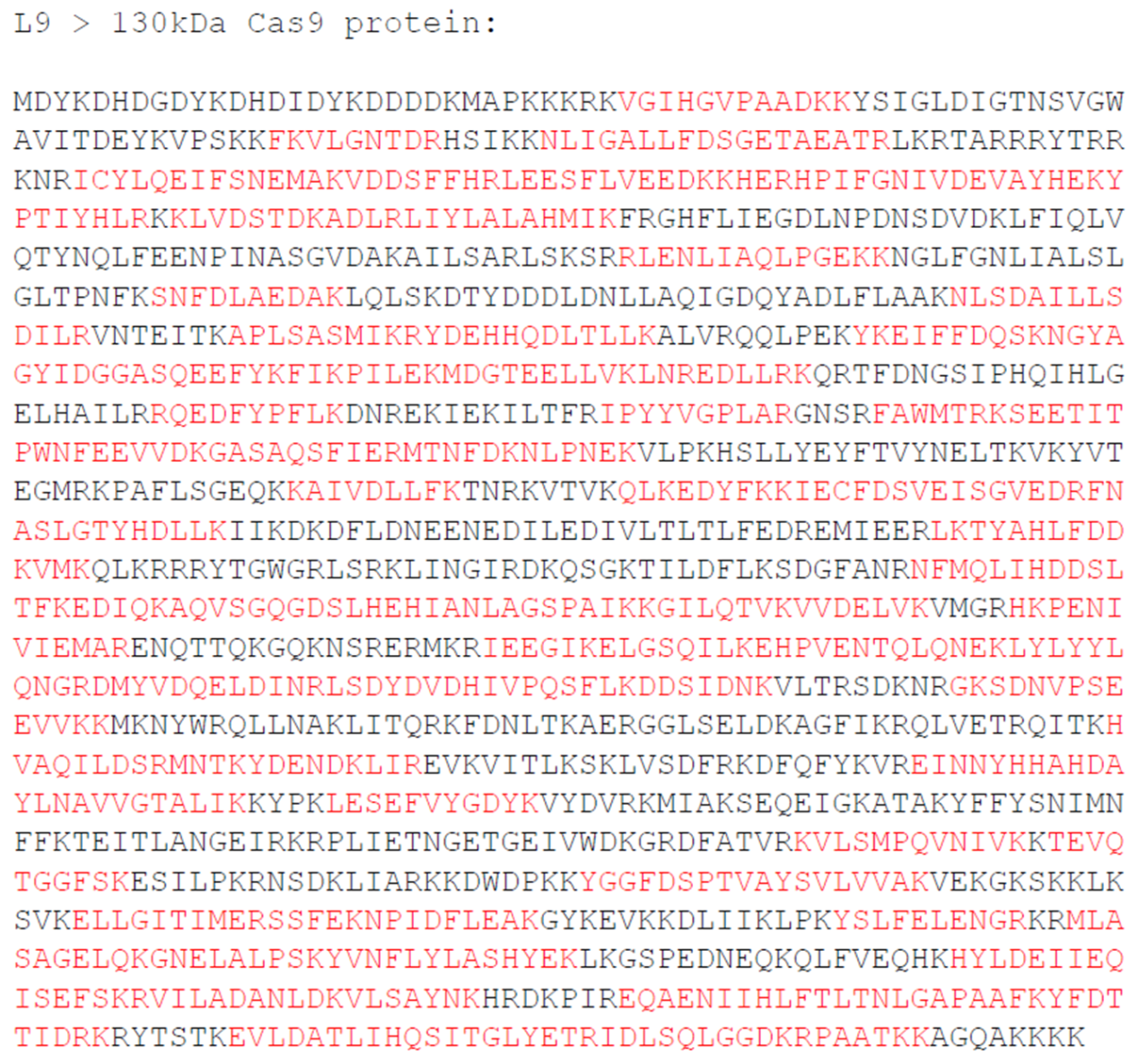


**Supplementary Figure S7.** Cas9 proteins (red and blue arrows) from Fig. 2 were identified by the LC-nESI-Q Exactive mass spectrometer. The protein regions covered by the fragments detected by the identification are labeled in red.


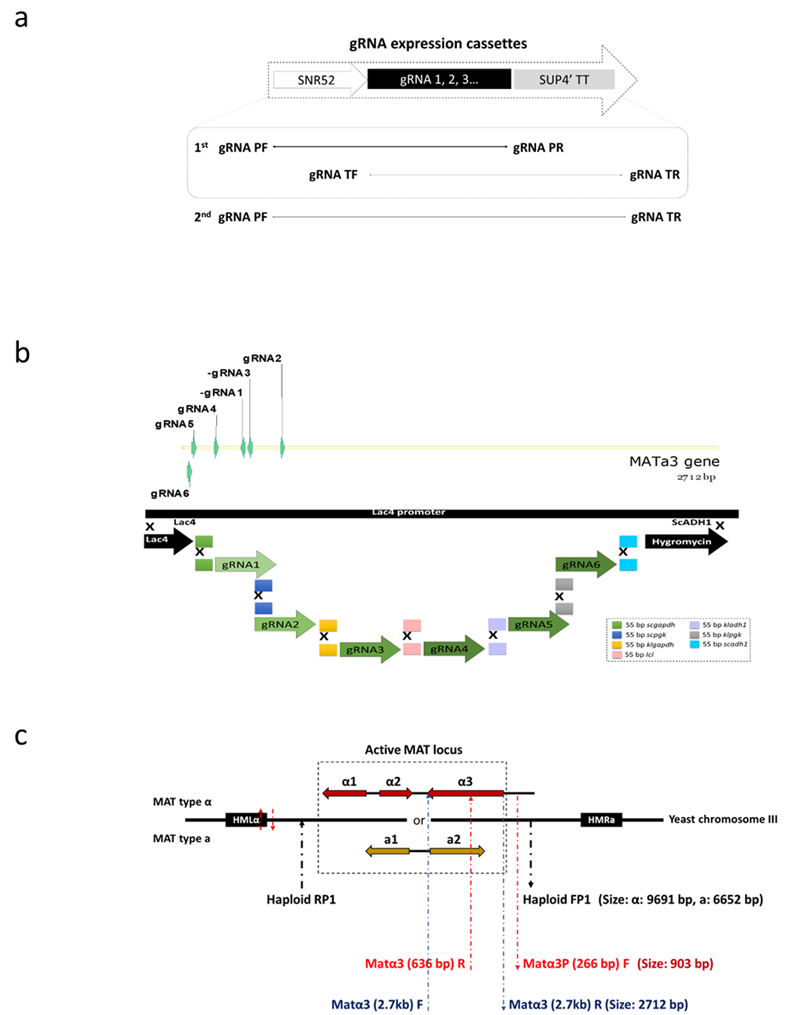


**Supplementary Figure S8.** Construction of PGASO cassettes. (a) The backbone of the yeast guide RNA (gRNA) expression cassette and the 20 bp inserting gRNAs of interests were directly synthesized into primer pairs and replaced by two-step fusion PCR. The backbone of the yeast guide RNA (gRNA) expression cassette with regulatory elements contains the SNR52 promoter, and the gRNA insertion region and the SUP4 terminator. The SNR52 promoter of gRNAs was amplified by PCR using the primer pair gRNA PF and gRNA PR, and the SUP4 terminator was amplified by the primer pair gRNA TF and gRNA TR. (b) The engineered yeasts were obtained via the application of a modified PGASO technique to assemble several gRNA cassettes in a predesignated order. Consecutive gRNA cassettes containing its specific overlapping 55 bp regions on the borders were used for recombining the cassettes. (c) Matα3 deletion was checked by PCR with two primer pairs of Matα3P(266 bp)-F x Matα3(636bp)-R and Matα3(2.7kb)-F x Matα3(2.7kb)-R; The MAT types of haploid and diploid were validated by the primer pair Haploid-FP1 and Haploid-RP1.


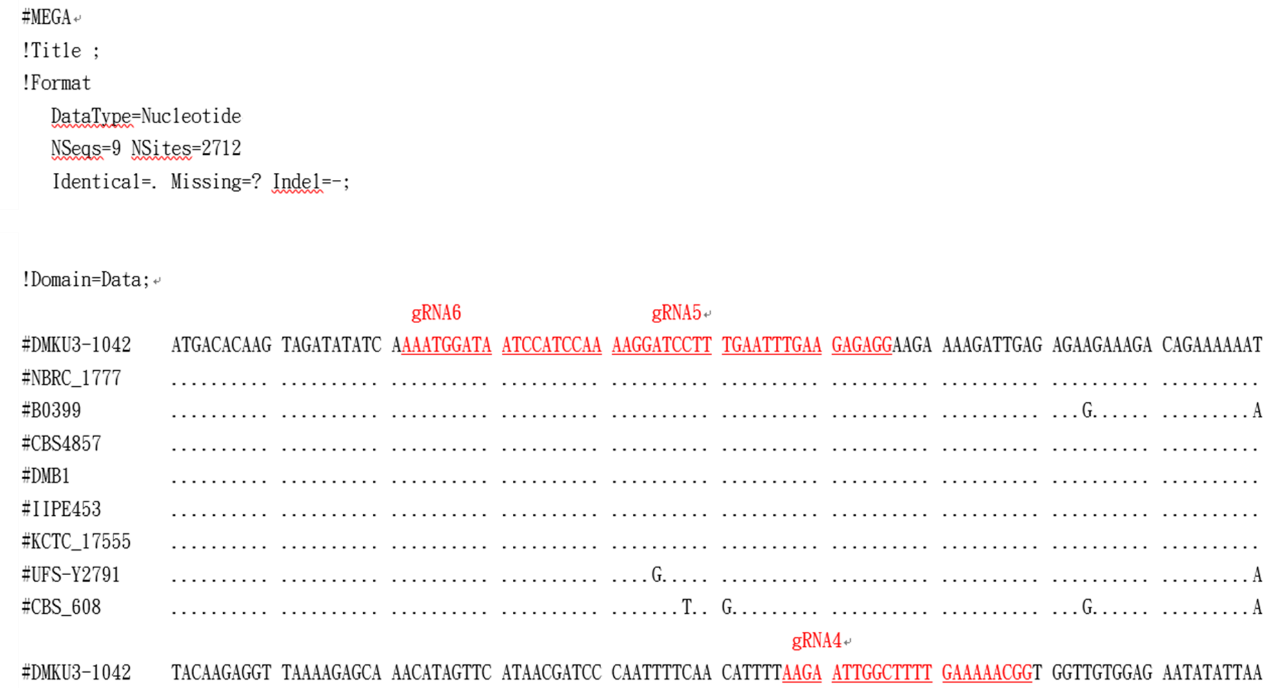


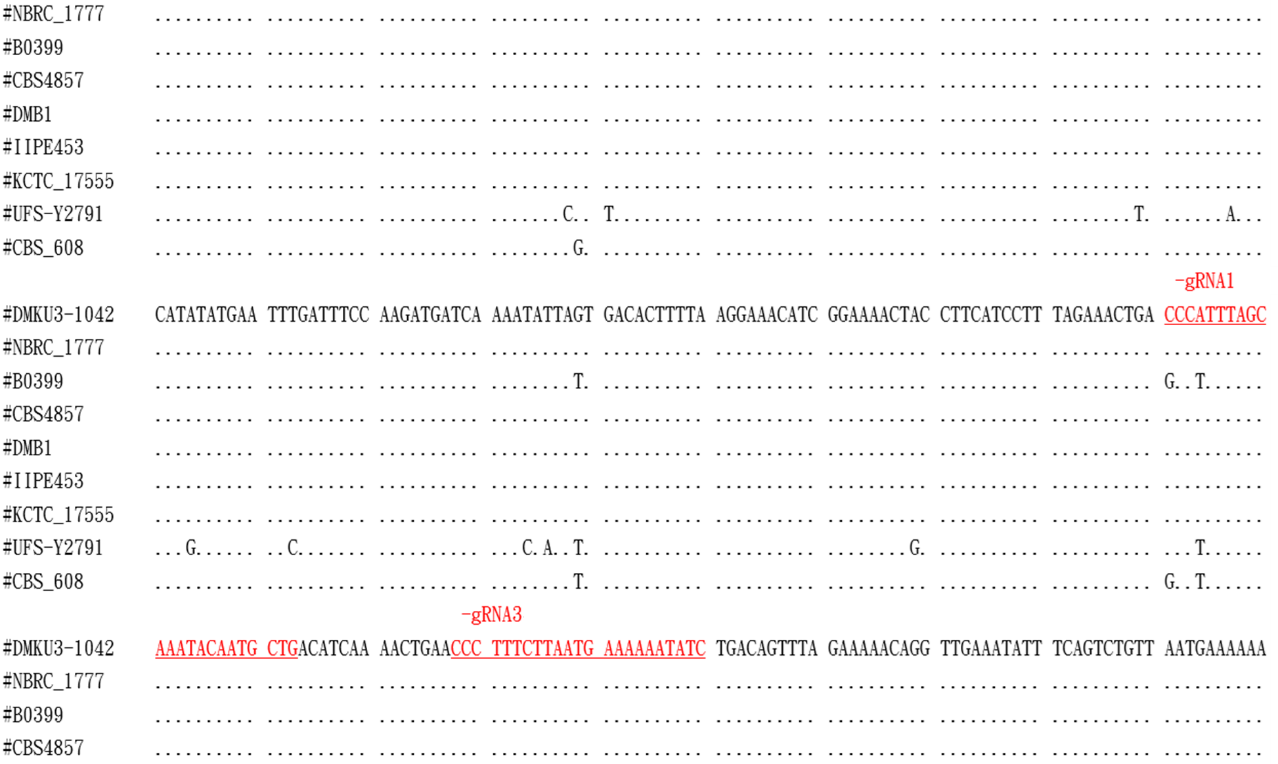


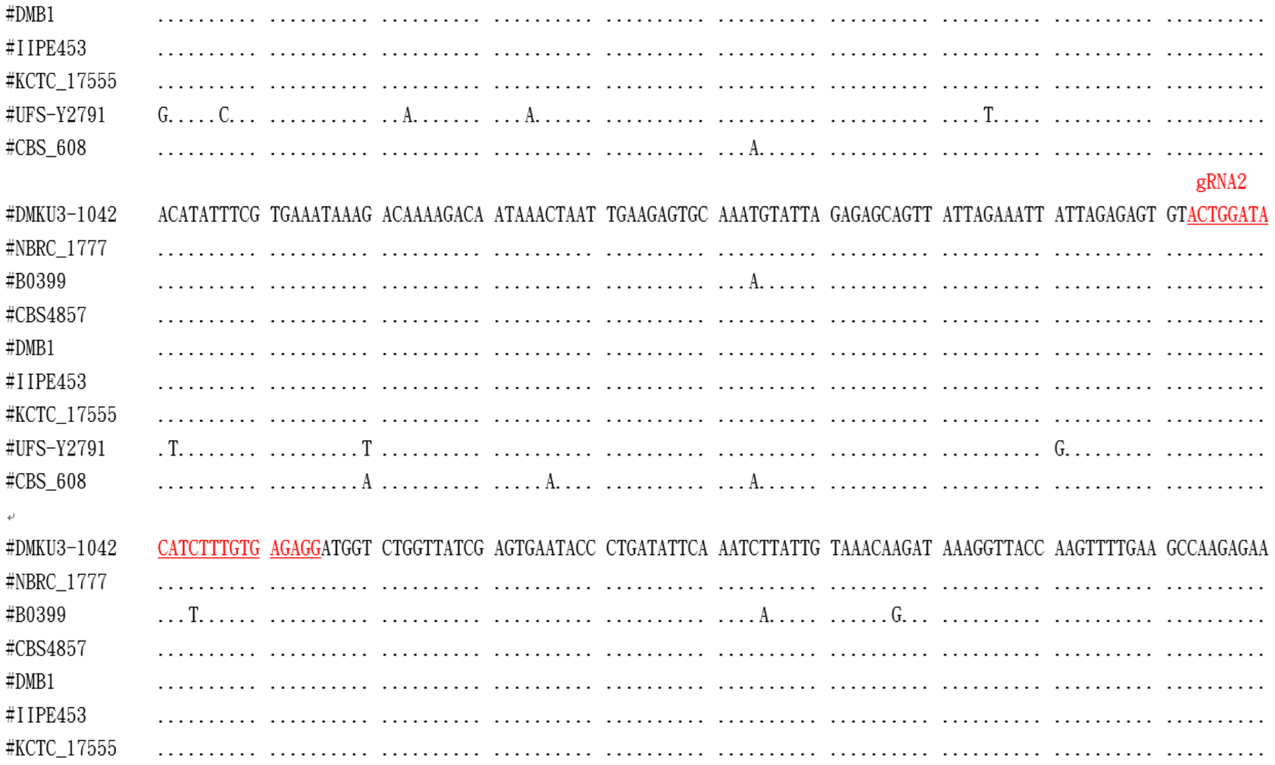


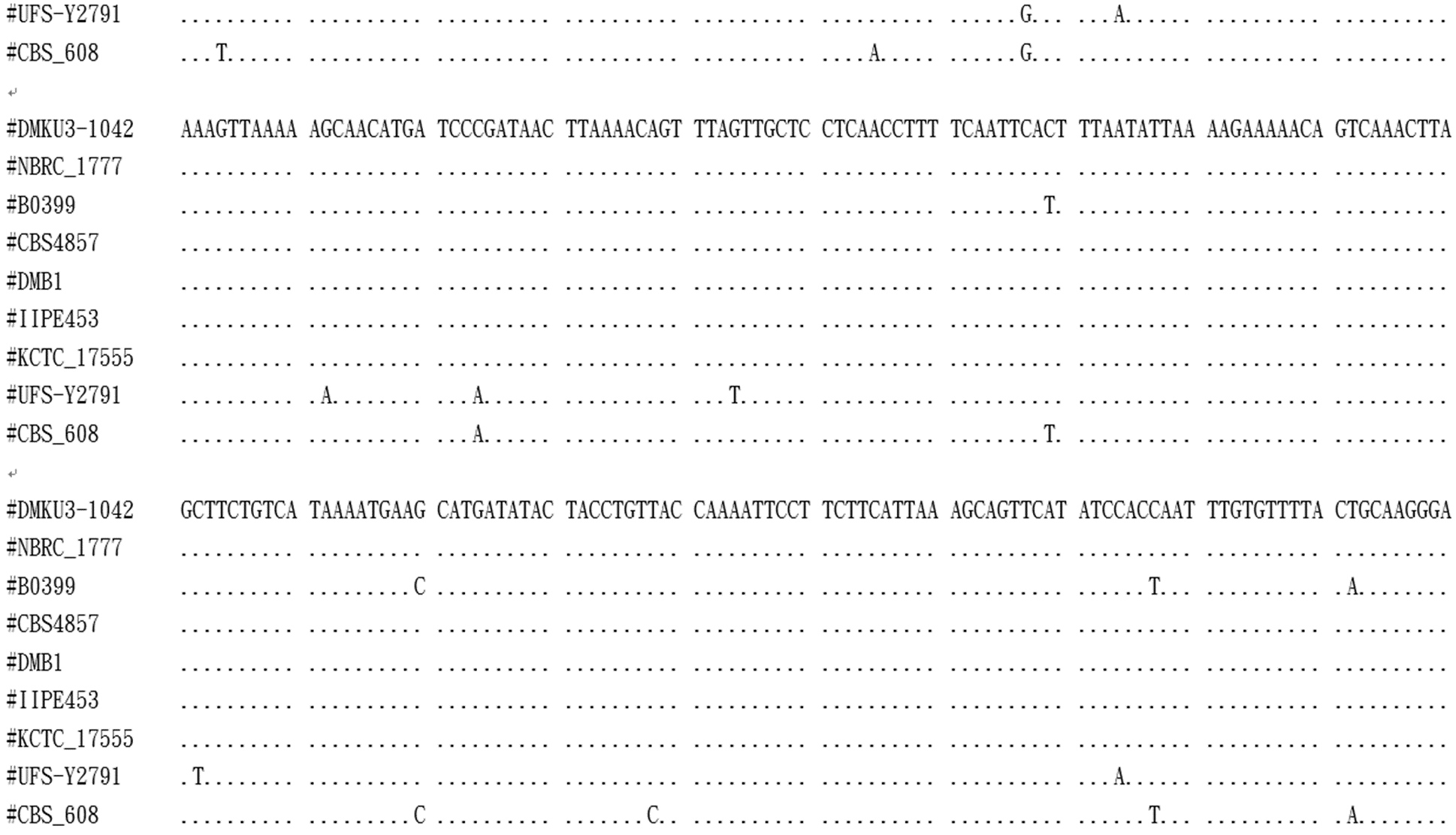


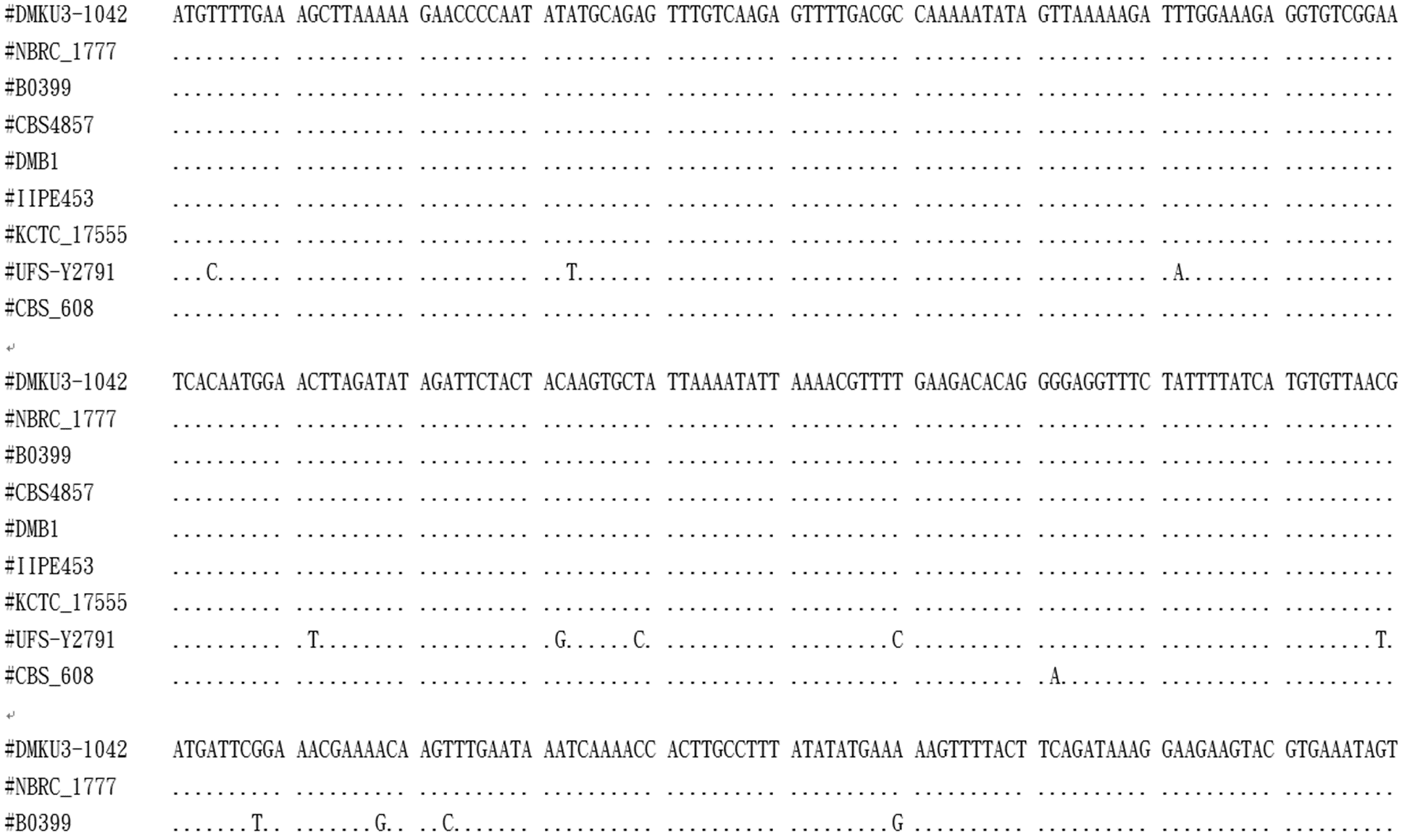


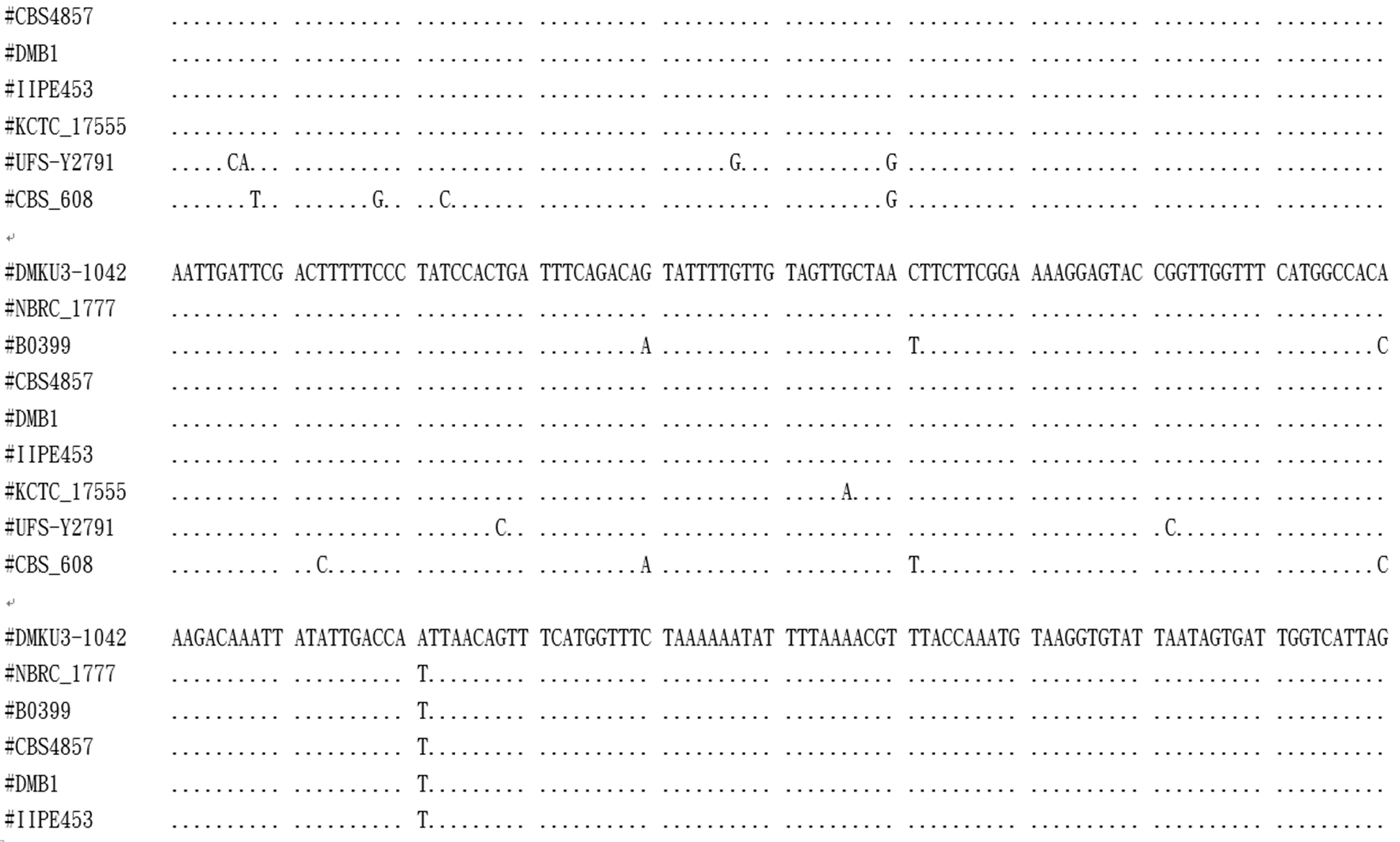


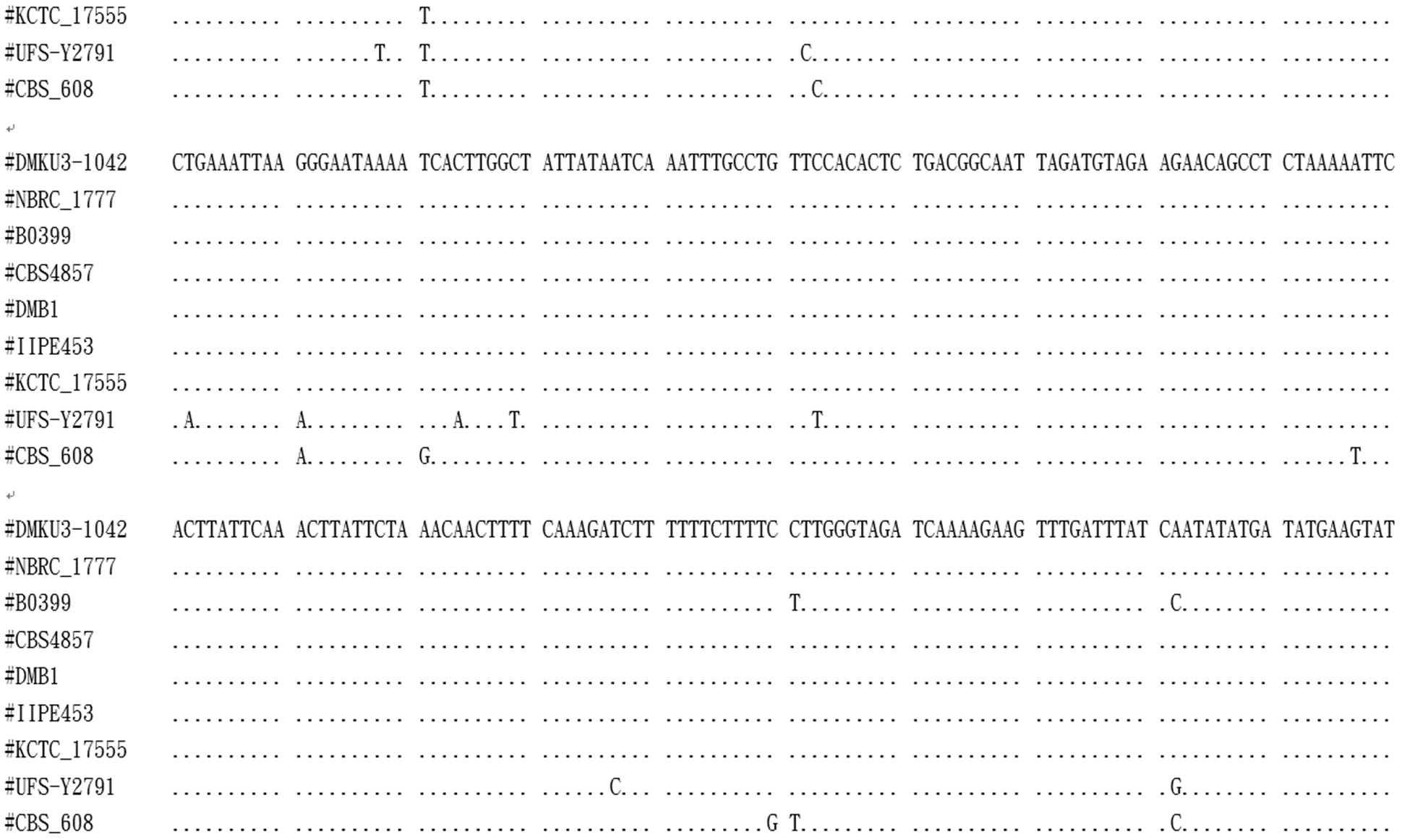


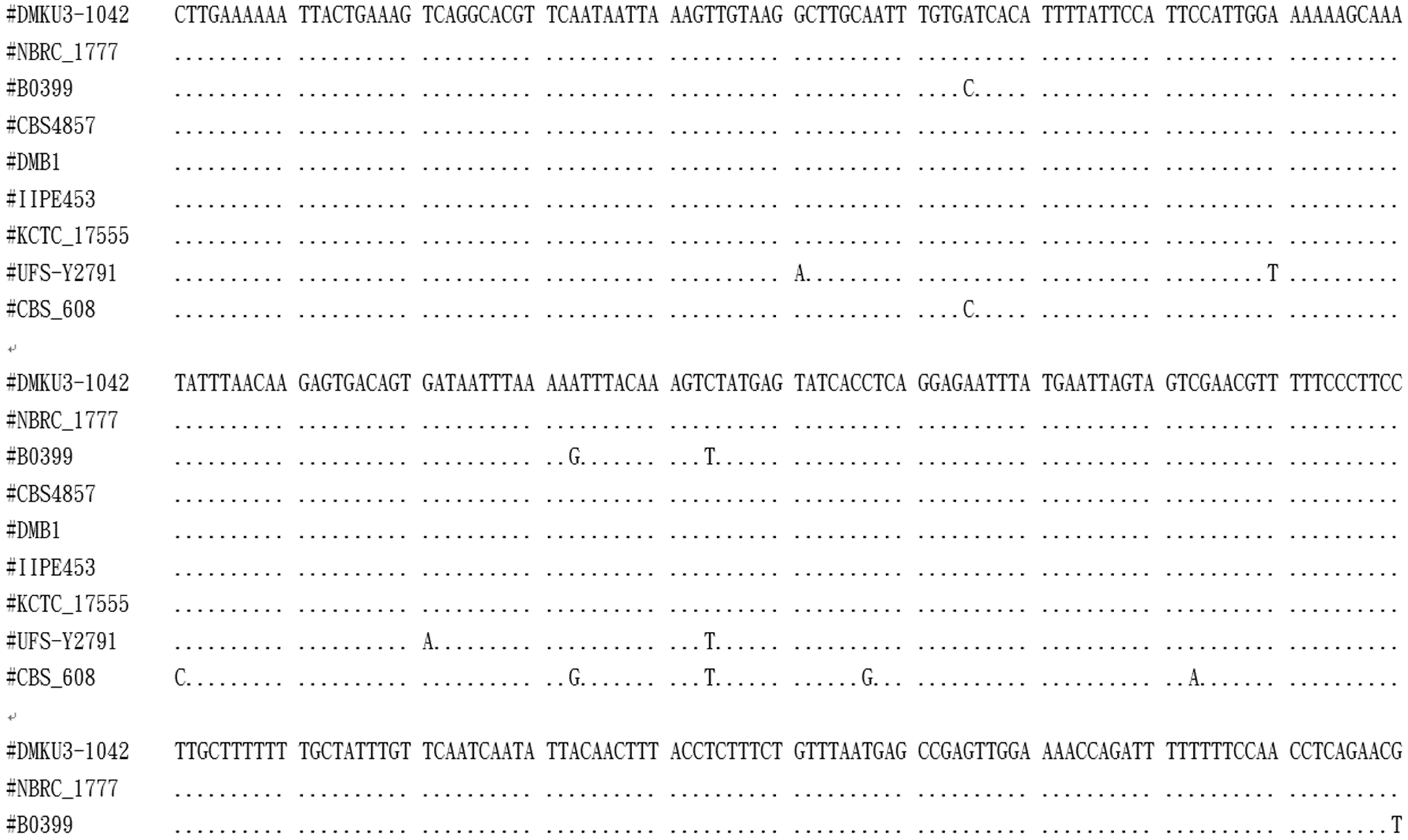


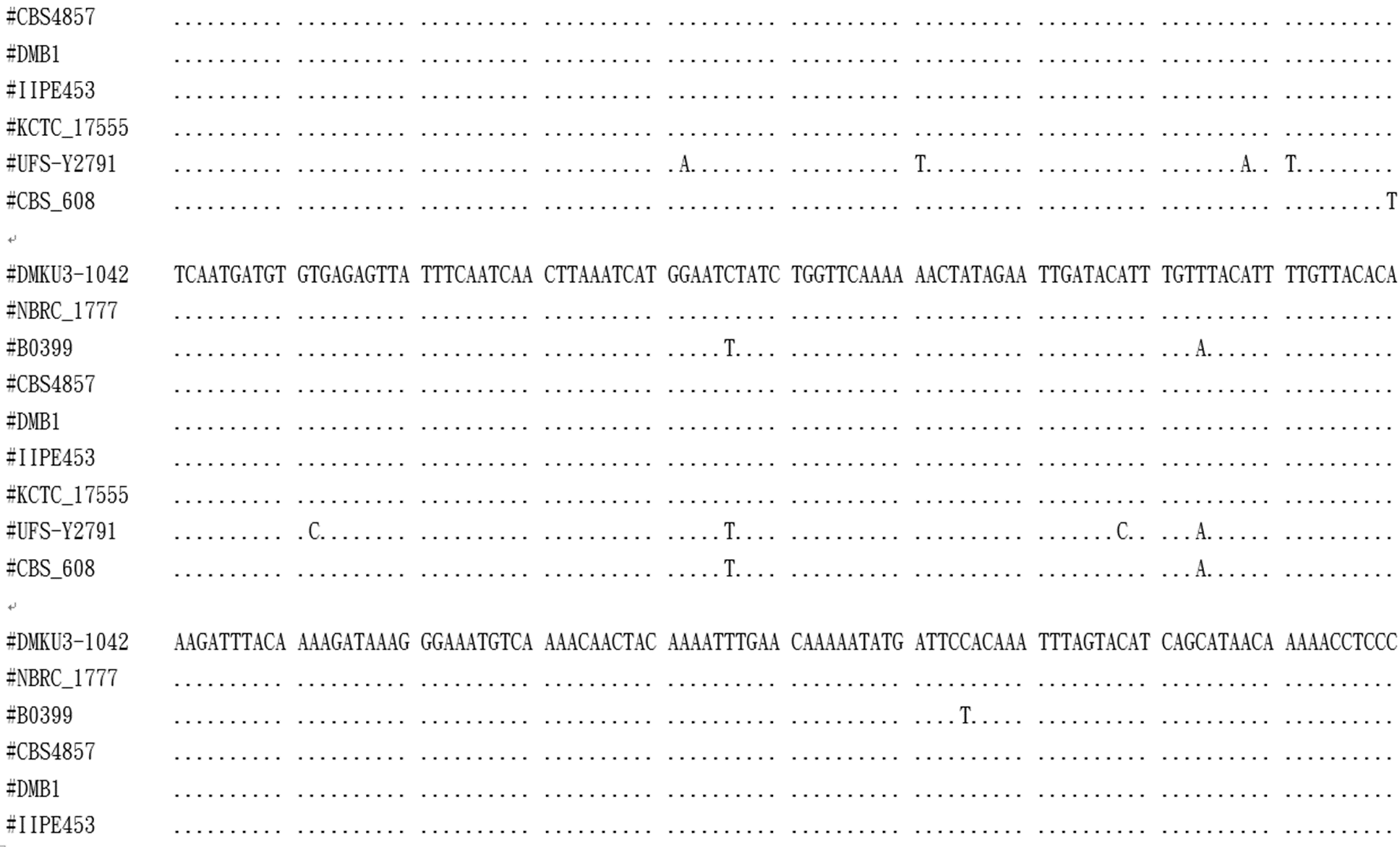


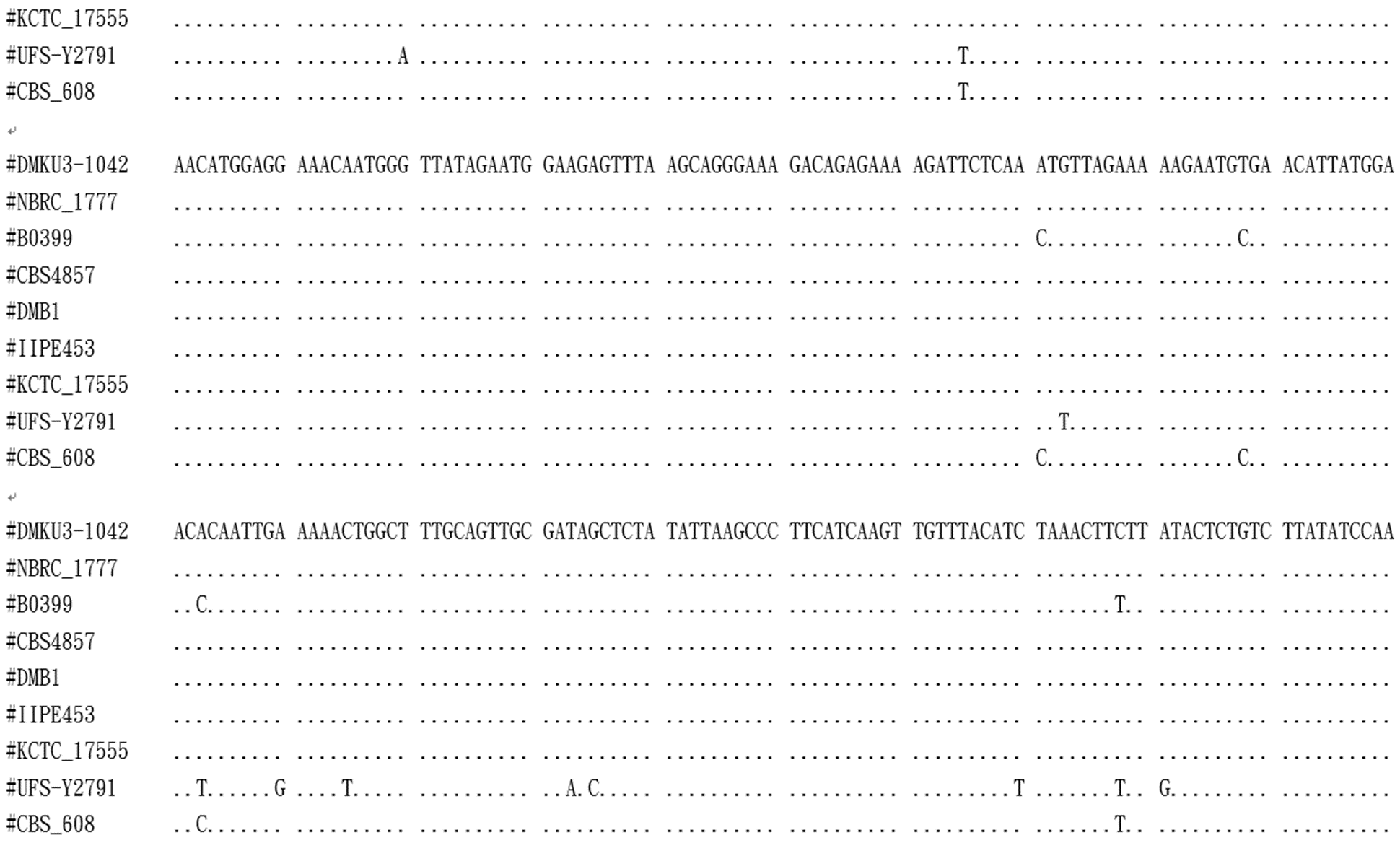


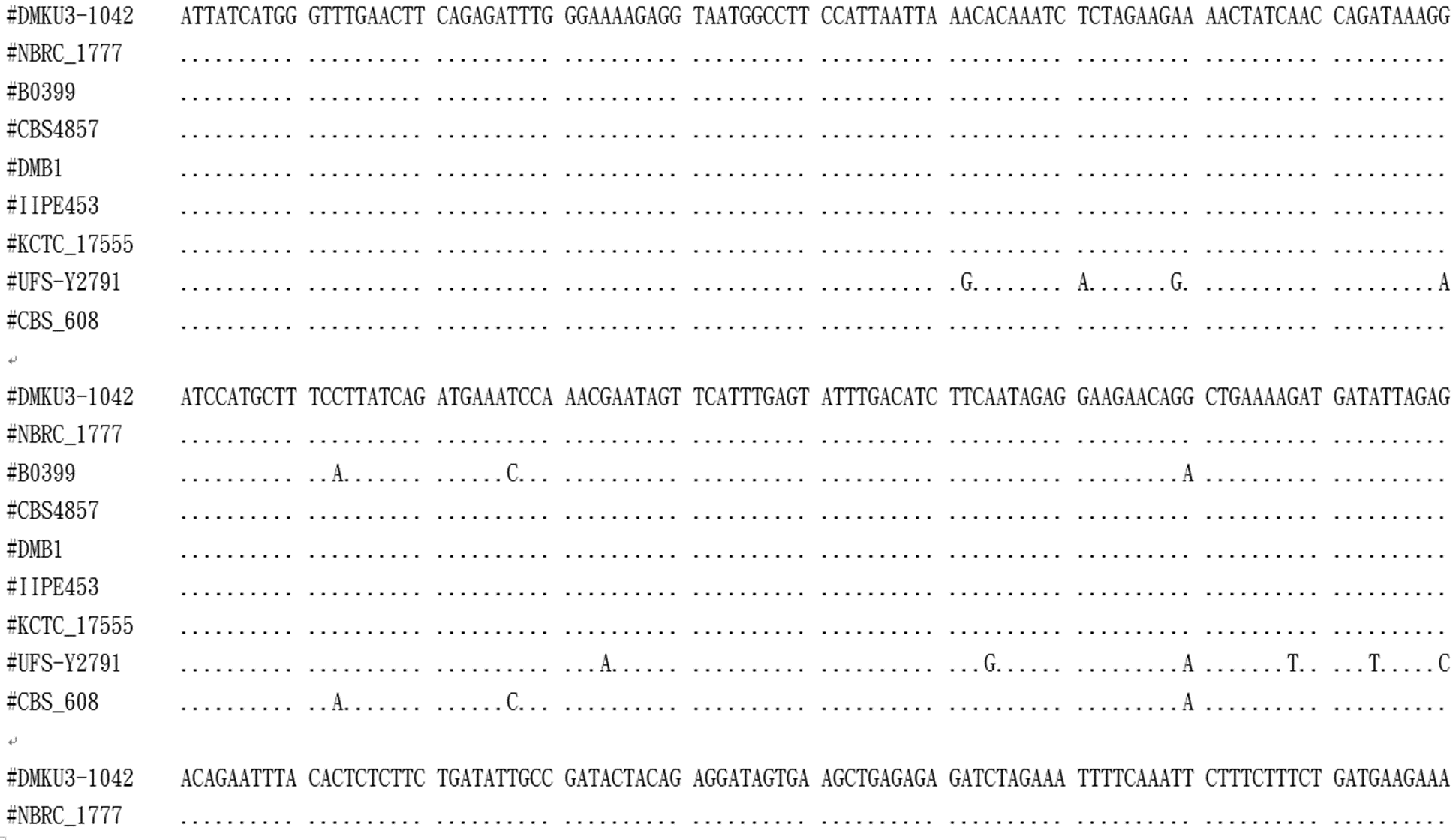


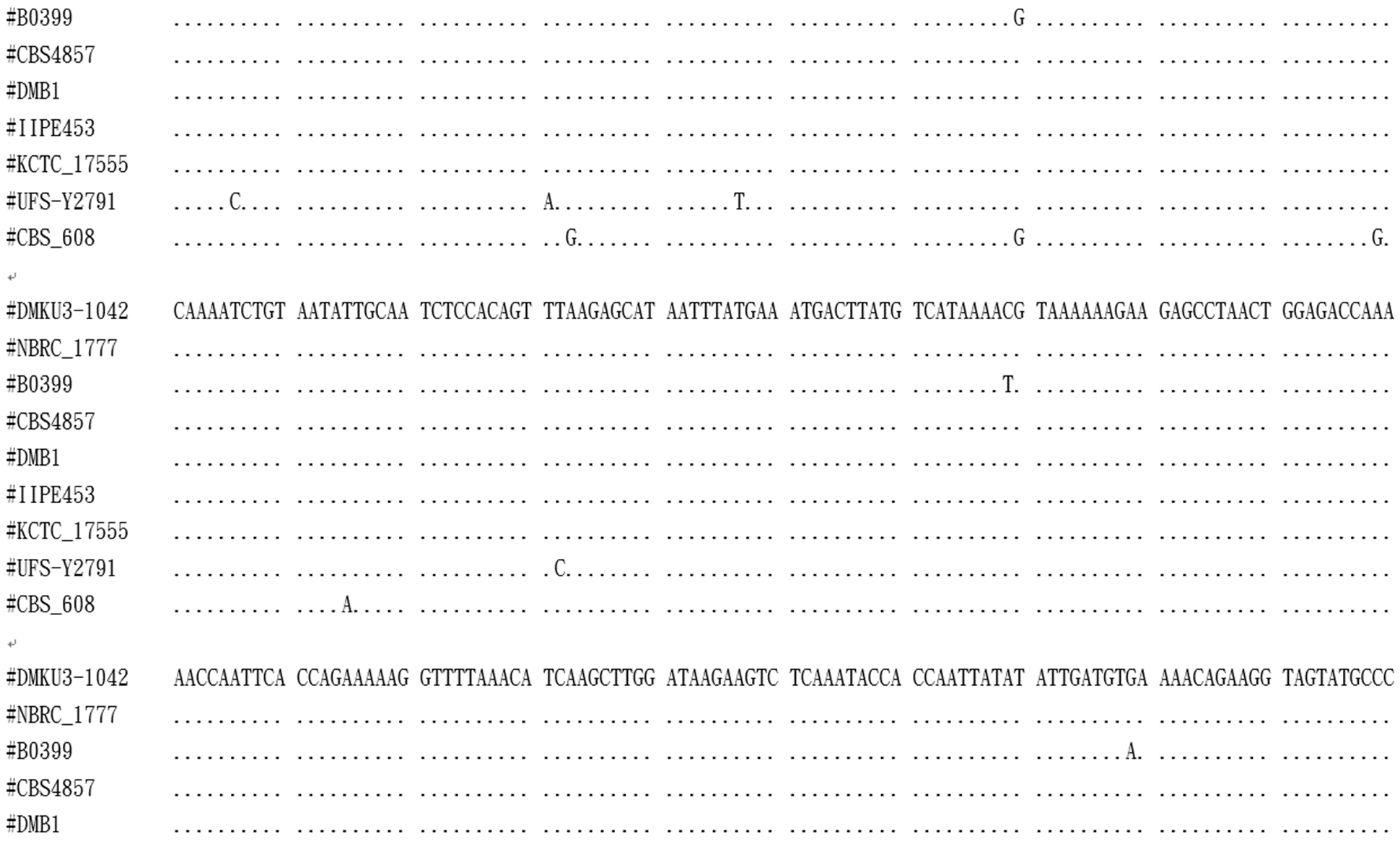


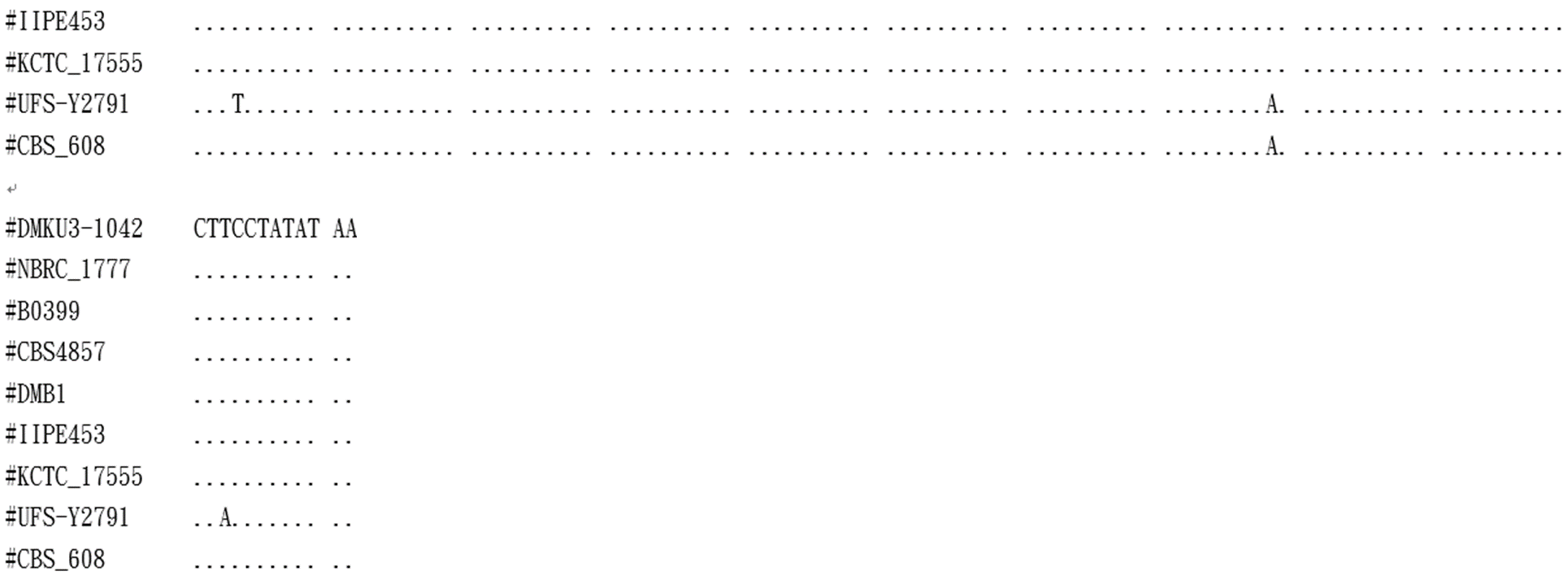


**Supplementary Figure S9.** Multiple sequence alignment of Matα3 gene in *K. marxianus* strains. The regions for the gRNA targets were labeled in red.


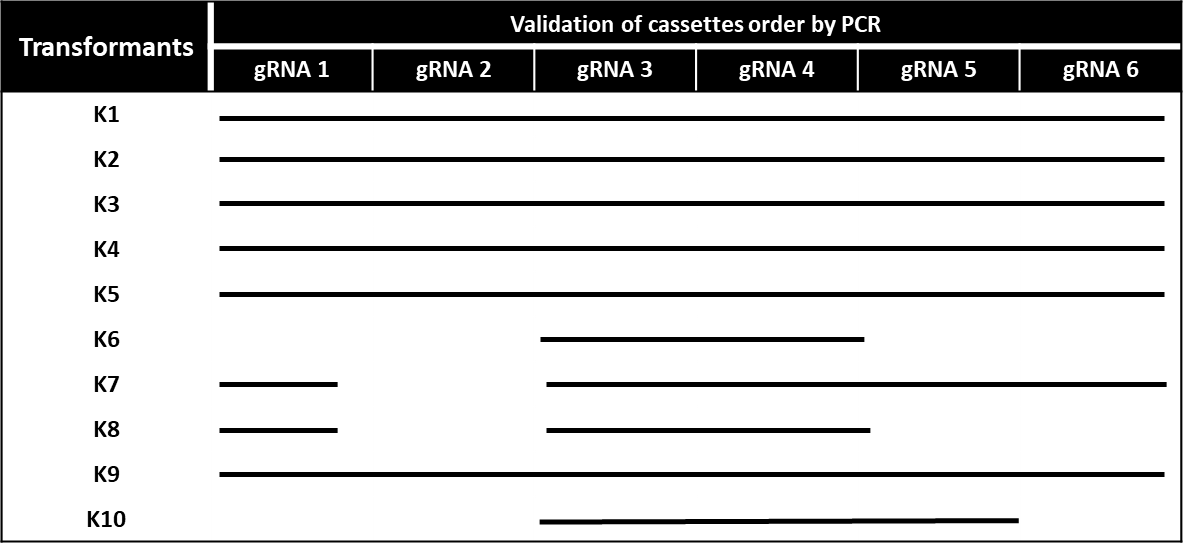


***Straight line means existence of the gRNA cassette**

**Supplementary Figure S10.** Validation of cassettes order by PCR. Six transformants (K1, K2, K3, K4, K5 and K9) contained all 6 gRNAs cassettes with the correct order, while the other four transformants only contained 2 to 5 gRNAs.


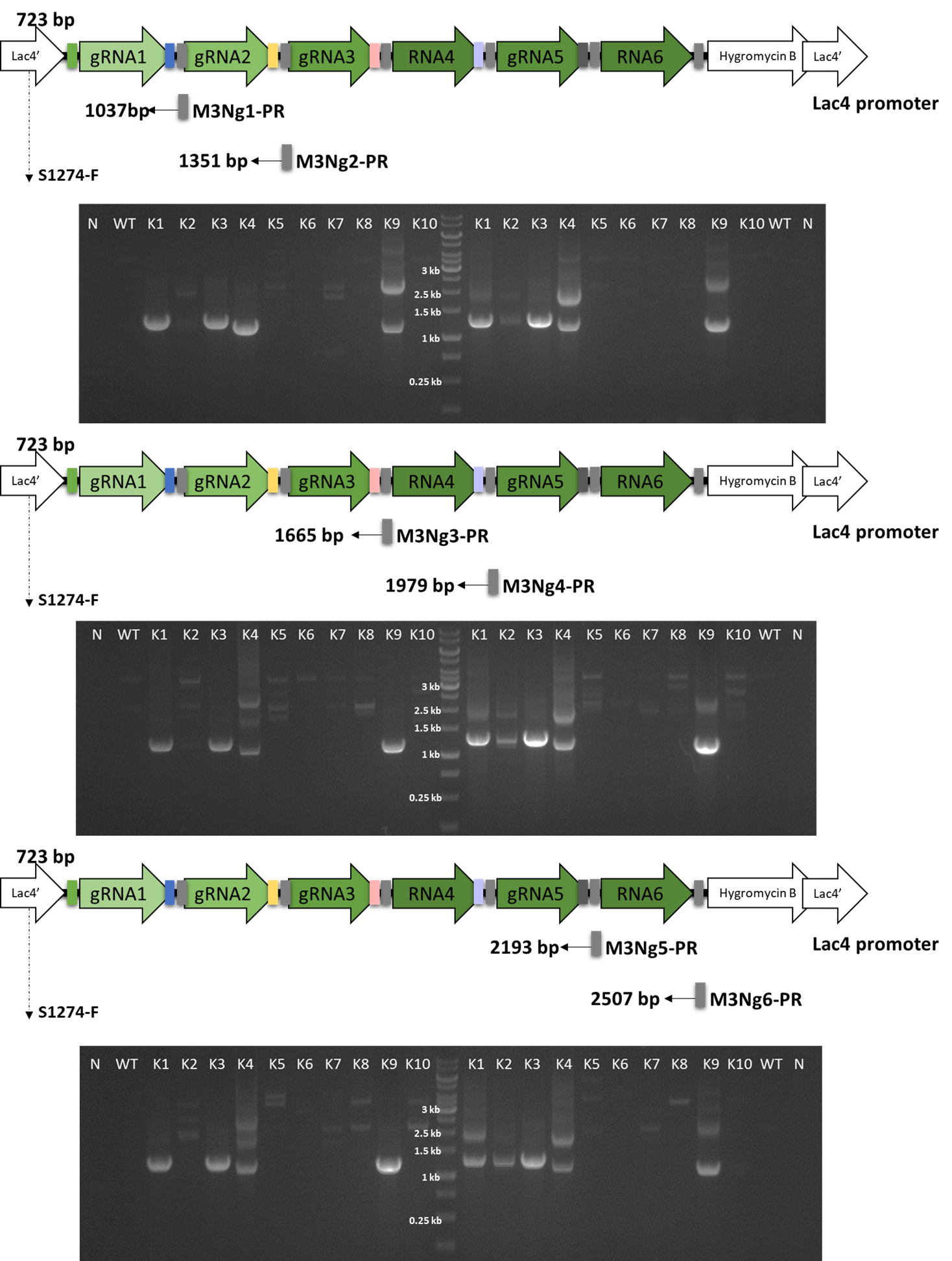


**Supplementary Figure S11.** Transformants with the correct assembled order of six integrated gRNA cassettes were confirmed by PCR.

**Supplementary Figure S12.** The successfulness of CRISPR/Cas9 cleavage on Matα3 gene in transformants K2, K3, K9 and K10 were validated by sequencing. N5 to N8 were only with point mutation on Matα3. Based on the location of indels, we inferred the gRNAs which facilitated the cleavage were gRNA2, gRNA4 and gRNA5.

**Supplementary Figure S13.** Sequencing result of pKLac4-Cas9-Zeocin plasmid by using the primers in Supplementary Table S6.

**Supplementary Figure S14** Standard curves for quantifying the expression of Cas9 protein via BSA (Bovine Serum Albumin).
